# Comparative transcriptomics between high and low rubber producing *Taraxacum kok-saghyz* R. plants

**DOI:** 10.1186/s12864-018-5287-4

**Published:** 2018-12-04

**Authors:** Francesco Panara, Loredana Lopez, Loretta Daddiego, Elio Fantini, Paolo Facella, Gaetano Perrotta

**Affiliations:** Trisaia Research Center, ENEA, Italian National Agency for New Technologies Energy and Sustainable Economic Development, MT, 75026 Rotondella, Italy

**Keywords:** Natural rubber, *Taraxacum kok-saghyz*, Russian dandelion, Transcriptomics

## Abstract

**Background:**

*Taraxacum kok-saghyz* R. (Tks) is a promising alternative species to *Hevea brasiliensis* for production of high quality natural rubber (NR). A comparative transcriptome analysis of plants with differential production of NR will contribute to elucidate which genes are involved in the synthesis, regulation and accumulation of this natural polymer and could help to develop Tks into a rubber crop.

**Results:**

We measured rubber content in the latex of 90 individual Tks plants from 9 accessions, observing a high degree of variability. We carried out de novo root transcriptome sequencing, assembly, annotation and comparison of gene expression of plants with the lower (LR plants) and the higher rubber content (HR plants). The transcriptome analysis also included one plant that did not expel latex, in principle depleted of latex transcripts. Moreover, the transcription of some genes well known to play a major role in rubber biosynthesis, was probed by qRT-PCR. Our analysis showed a high modulation of genes involved in the synthesis of NR between LR and HR plants, and evidenced that genes involved in sesquiterpenoids, monoterpenoids and phenylpropanoid biosynthesis are upregulated in LR plants.

**Conclusions:**

Our results show that a higher amount of rubber in the latex in HR plants is positively correlated with high expression levels of a number of genes directly involved in rubber synthesis showing that NR production is highly controlled at transcriptional level. On the other hand, lower amounts of rubber in LR plants is related with higher expression of genes involved in the synthesis of other secondary metabolites that, we hypothesize, may compete towards NR biosynthesis. This dataset represents a fundamental genomic resource for the study of Tks and the comprehension of the synthesis of NR and other biochemically and pharmacologically relevant compounds in the *Taraxacum* genus.

**Electronic supplementary material:**

The online version of this article (10.1186/s12864-018-5287-4) contains supplementary material, which is available to authorized users.

## Background

Natural rubber (NR) is a biopolymer mainly composed of poly (cis-1,4–isoprene) synthesized in the latex of many plant species. At present, the only commercial source of NR is the rubber tree (*Hevea brasiliensis* Muell. Arg., Hb); however, several aspects like vulnerability to pathogens, reduced genetic diversity, long juvenile phase, and restriction only to tropical environments, make it necessary to search for additional NR crops. In the last decades, a renewed interest in Russian dandelion (*Taraxacum kok-saghyz* Rodin, Tks) as an alternative rubber producing plant, has raised thanks to the high quality of its NR [1]. Tks is a small diploid, sexually reproducing, rosette shaped plant with specialized cells, known as laticifers, that form continuous channels by the entire or partial dissolution of the end walls [2, 3]. Latex, the cytosol of laticifer cells, is whitish and rich in proteins and secondary metabolites and it is more abundant in the roots. Novel NR is continuously produced and stored in the rubber particles (RP) that are small, globular vesicles, surrounded by a lipid monolayer membrane and dispersed in the latex [4].

The basic building block of NR is isopentenyl pyrophosphate (IPP), mainly produced via the mevalonic acid (MVA) pathway [5]. The same pathway also provides IPP for the synthesis of many other isoprenoid end-products, including monoterpenoids, sesquiterpenoids, triterpenoids, steroids and others that fulfil important roles in plant growth, development and protection against phytopathogens and herbivores [6].

Rubber chain elongation is mediated by sequential addition of IPP monomers to an allylic pyrophosphate initiator, such as farnesyl pyrophosphate [7]. This reaction is catalysed by a specialized rubber cis-prenyltransferase (CPT), that forms a major component of the rubber transferase (RTase) complex on the surface of RPs, depositing nascent rubber chains into the particle core [8, 9].

The recent publication of a draft Tks genome showed the presence of 8 CPTs. TkCPT 1–4 are likely involved in rubber elongation as they belong to the same phylogenetic group of *HbHRT1* and *HbHRT2* [10–12].

CPTs do not form RPs and do not produce high-molecular-mass rubber alone [4, 13–16]. The RTase complex contains other proteins that are necessary for full catalytic activity. Recently, in Hb, a protein complex that functions as NR biosynthetic machinery has been described. It consists of HbHRT1 and two RP-bound proteins, HbREF and HbRBP. The latter is a homologue of the human Nogo-B receptor (NgBR), also named CPT-like, that interacts specifically with HRT1 and it is a key factor for HRT1 to show RTase activity [11].

In Hb the two most abundant RP proteins, rubber elongation factor (REF) and small rubber particle protein (SRPP), have been shown to influence cis-polyisoprene production [10].

Also in *Taraxacum brevicorniculatum* (Tb), a close relative of Tks, NR is synthesized on the surface of RPs, which are stabilized by auxiliary proteins such as TbSRPPs and the rubber elongation factor (TbREF) [17].

TbSRPPs family is composed by 5 members; among them, TbSRPP3, 4 and 5 are the most abundant on RPs and play an important role in NR efficient synthesis [18]. Although not absolutely required for rubber biosynthesis, TbREF is able to stabilize RPs and to enhance NR production [17]. Also in Tb a NgBR homolog, TbRTA, is an essential component of the RTase complex and its knockdown heavily impaired NR biosynthesis [19]. In Tks genome one TkREF, nine TkSRPPs and two TkCPT-like genes have been found [12]. Three RP proteins have been investigated in Tks: TksSRPP3, 4 and 5 and, the most abundant, TksSRPP3, has a role in determining both NR quantity and quality [20].

Roots of *Taraxacum spp*, beside NR, are rich in the storage carbohydrate inulin and various classes of specialized metabolites relevant for their biochemical and pharmacological potential [21]. Inulin is a polysaccharide belonging to the class of fructans and is mainly composed of fructose units. In Tks, inulin ranges from ~ 10 to ~ 50% of root dry-weight biomass [22]. Degradation of inulin is considered the main source of glycolysis intermediates to produce novel Acetyl-CoA that, in turn, is used to feed MVA and NR production. In both Tks and Tb, the overexpression of *fructan 1-exohydrolase* (*1-FEH*), the key enzyme in inulin degradation, resulted in plants with doubled rubber content in the roots [23].

Relevant secondary metabolites of dandelion roots are the sesquiterpene lactones mostly of the eudesmanolide and germacranolide types. Dandelions also contain triterpenes, phytosterols, several phenylpropanoids, various phenolic acids (for example, chicoric, monocaffeoyltartaric, chlorogenic, and caffeic acids) and coumarins in various amounts [24, 25].

Tks germplasm was recently recollected by USDA [26] and within the EU-PEARLS project initiative [27]. Natural populations characterized so far, show a high degree of variability in terms of rubber content [22, 28]. To make Tks a profitable rubber crop, one of the aspects that should be considered by breeders is obtaining genotypes with higher and stable rubber concentrations [29]. A better understanding of rubber biosynthesis and its control in Tks, as well as the identification of rate-limiting enzymes and the competing metabolic pathways, is necessary to define both classical and biotechnological breeding strategies. Metabolic engineering in this species is possible as Tks is amenable to genetic manipulation [30, 31].

In the present study, to identify plants with contrasting ability in synthesizing rubber, we determined NR content in the latex at 9 months age in about 90 individuals taken from 9 different Tks accessions. Afterward we analysed root gene expression in plants with extreme phenotypes. Transcriptomic experiment has been carried out by a 454 pyrosequencing approach. Data analysis enabled us to better comprehend the mechanisms underpinning such different ability in the synthesis of NR and to identify which genes and pathways are involved.

## Results and discussion

### Differential rubber production in Russian dandelion accessions

**Fig. 1:**
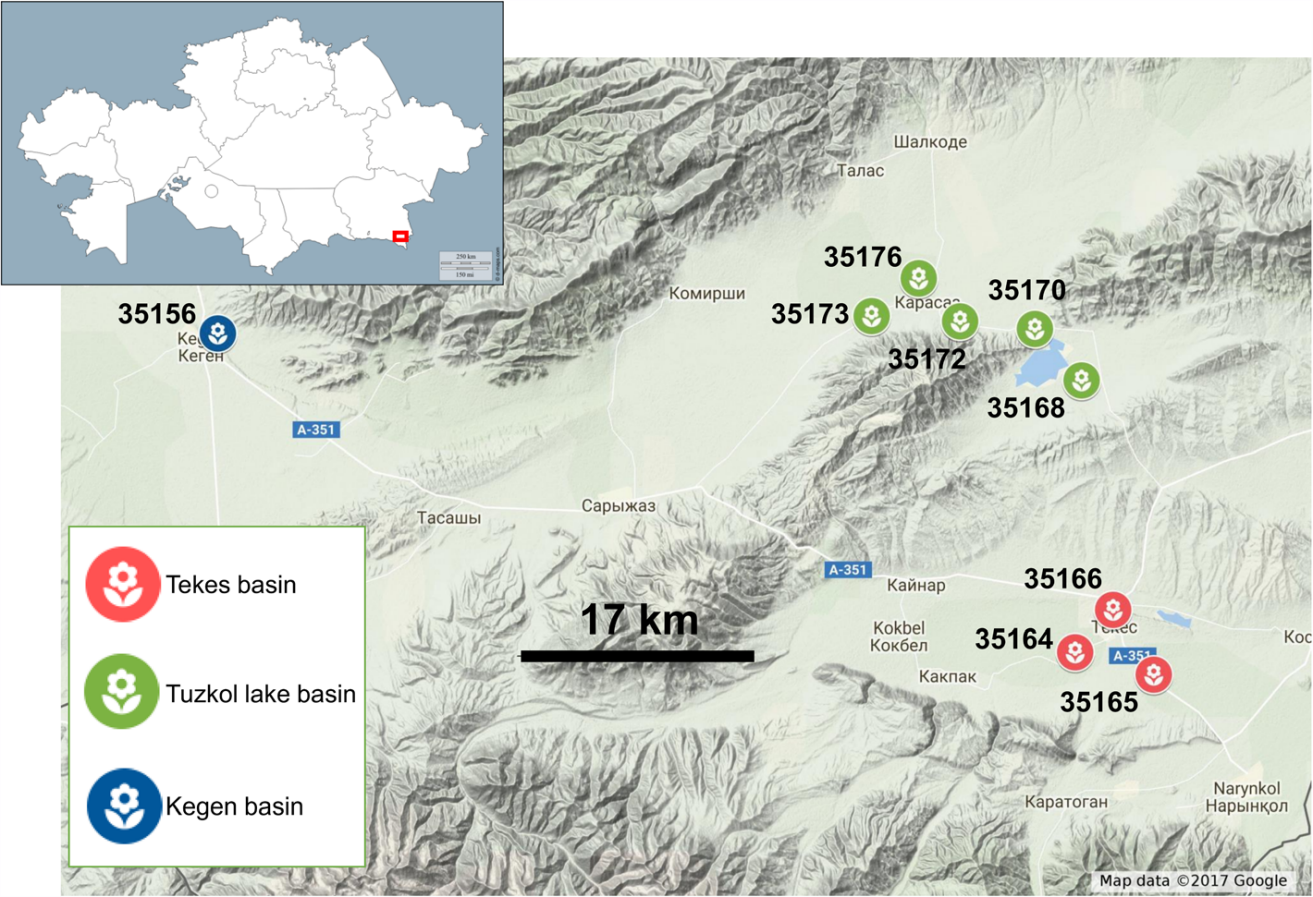
Map indicating the collection site of the nine Tks accessions analyzed in the present study. On the top-left corner is represented the map of Kazakhstan (modified from http://d-maps.com). The red-boxed region in the bottom-right corner is the sampling area that is represented below at higher scale (made with Google maps web application). Collection sites are indicated with flower shaped icons

**Fig. 2:**
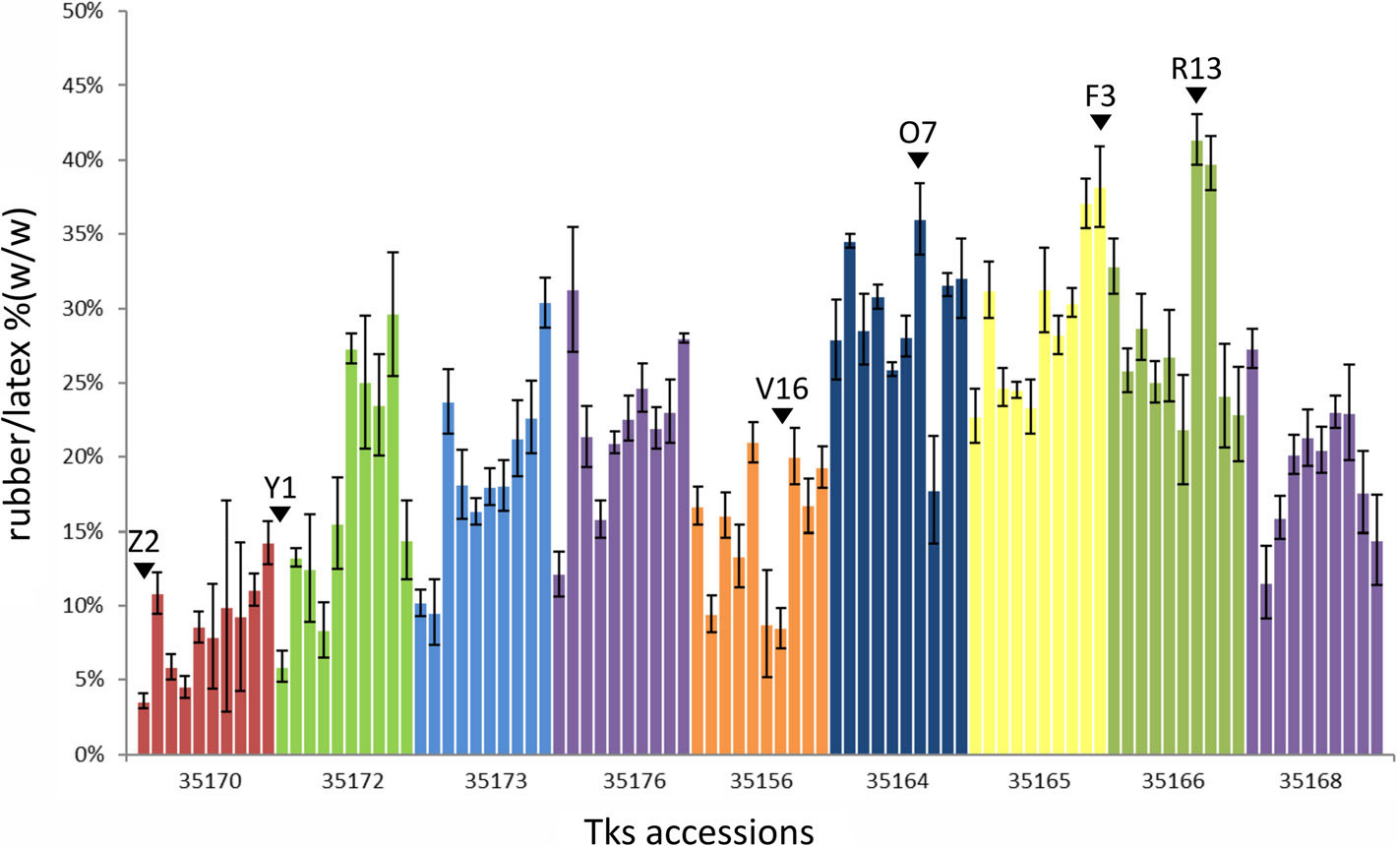
Rubber content (% *w*/w) of the plants analyzed in this paper. Different colors represent the diverse accessions. HR and LR plants used in the transcriptomic experiments are indicated by black arrowheads

To identify plants that, at the same conditions, were differentially active in NR synthesis, the rubber content in the latex, expressed as percentage of rubber/latex (% *w*/w) was evaluated. Nine months old Tks plants were used as they were expected to be actively synthesizing NR at that time [32, 33]. Nine accessions of Tks collected in three different sites (Fig. 1) were used and, for each accession 10 individuals were analysed. Results of rubber analysis are shown in Additional file 1, Table S1 and Fig. 2 as individual plants and in Table 1 and Additional file 2: Figure S1 as accessions.

**Table 1:** Results of separation of means using Tukey’s Honest Significant Difference (HSD) test

Accession	LSM	SD	Tuckey groups	A	B	C	D	E	Collection site
35164	29.33	0.051135	AB	*	*				Tekes basin
35165	29.18	0.054969	AB	*	*				Tekes basin
35166	28.93	0.068861	AB	*	*				Tekes basin
35176	22.19	0.054454	ABCD	*	*	*	*		Tuzkol lake basin
35168	19.48	0.044176	BCD		*	*	*		Tuzkol lake basin
35173	18.83	0.062130	BCD		*	*	*		Tuzkol lake basin
35172	17.54	0.082370	BCD		*	*	*		Tuzkol lake basin
35156	14.99	0.047366	BCDE		*	*	*	*	Kegen basin
35170	8.58	0.032493	DE				*	*	Tuzkol lake basin

Levels connected by same letter are not significantly different. *LSM* Least Square Mean of rubber/latex (*w*/w %). *SD* Standard Deviation

The average rubber content was 21% rubber/latex, ranging from 41% of R13 plant (accession 35166) to 3.6% of Z2 (accession 35170). The populations with the highest and the lowest rubber amount were 35164 (29% rubber/latex) and 35170 (8.6% rubber/latex), respectively.

Three more sampled plant individuals, Z1 (from accession 35170), S2 (from accession 35165), and Y11 (from accession 35172), could not be included in this analysis as they expelled very little (Z1, S2) or no latex (Y11), from excised roots.

In a previous study based on SSR markers on a panel of populations including those used in our work, authors observed low average level of differentiation amongst populations and no signature of isolation-by-distance [28]. Nevertheless, as shown in Table 1, accessions with the highest rubber content were collected in the same area, the Tekes basin, giving a hint about a possible population structure for rubber production.

### Sequencing and assembly

To correlate rubber content to the genetic background of the assayed samples, we generated a root transcriptome from three plants with High Rubber (HR) content (R13, F3, O7), three plants with Low Rubber (LR) content (Y1, V16, Z2) and one plant with no collectable latex (Y11). HR and LR individual plants were selected from different accessions to minimize population-related variations. The Y11 plant was considered in the transcriptomic survey because of the absence of latex, where the synthesis of rubber occurs, could, in principle, provide new insights about transcription alterations of rubber production related genes.

Three shotgun cDNA libraries from HR, LR and Y11 plants were sequenced using 454 GS FLX + Titanium Sequencer (Roche Diagnostics Corporation, Switzerland); a total of 185,163, 186,148 and 172,263 raw sequence reads were generated (Additional file 1: Table S2). After trimming and filtering of raw sequences, 523,876 high-quality reads from all samples, were obtained and then assembled by using GS De Novo Assembler Software (Roche Diagnostics Corporation, Switzerland). About 83% of the sequenced reads were assembled in 16,416 unique contigs with an N50 of 1351 bp, while the remaining unassembled reads (62,184) were classified as singletons. The size of the assembled transcripts was approximately 253 Mb, with fragment lengths ranging from 100 to 8079 bp (Additional file 1: Table S3). Transcript assemblies with a minimal length of 200 bp were deposited to DDBJ/EMBL/GenBank under the project accession GFZW00000000.

### Functional annotation

To identify putative gene functions, unigenes were annotated using stand-alone NCBI Blast software (mpiblast 1.6.0) against nr (NCBI non-redundant protein), TAIR (The Arabidopsis Information Resource) and UniprotKB/SwissProt (a manually annotated and reviewed protein sequence database) current public databases (e-value ≤1e^− 5^). GO (Gene Ontology) annotation and KEGG (Kyoto Encyclopedia of Genes and Genomes) classification were also performed by Blast2GO PRO [34] and Kobas 3.0 [35] softwares (Additional file 1: Table S4).

Of 73,350 unigenes, 50,383 (69%) were annotated in at least one of the above mentioned public databases (Additional file 1: Table S4, S5). Among annotated unigenes, 26,538 out of 73,350 (36%) were assigned to one or more GO term annotations [36] (Additional file 1: Table S4). The most represented terms were: ‘Metabolic Process’ and ‘Cellular Process’, within Biological Process category; ‘Membrane’, ‘Cell’, and ‘Cell Part’ within the Cellular Component category; ‘Catalytic Activity’ and ‘Binding’ within Molecular Function category (Additional file 2: Figure S2).

Furthermore, 4840 sequences were assigned to an Enzyme Commission (EC) number, categorized into the six classes: Oxidoreductases (774), Transferases (1546), Hydrolases (1994), Lyases (224), Isomerases (202) and Ligases (216) by Blast2GO PRO (Additional file 2: Figure S3).

Finally, to identify enzymes involved in the main biological pathways, gene transcripts were assigned to canonical pathways in the KEGG database (http://www.genome.jp/kegg/).

From this analysis, we identified 11,096 transcripts (15%) assigned to 129 metabolic pathways, grouped in the five main KEGG categories: “metabolism”, “genetic information processing”, “environmental information processing”, “cellular process”, and “organismal systems” articulated in 18 sub-categories (Additional file 2: Figure S4 and Additional file 1: Table S6). Oxidative phosphorylation, starch and sucrose metabolism, purine metabolism, glycolysis/gluconeogenesis, pyruvate metabolism and pyrimidine metabolism were the most represented pathways (Additional file 1: Table S6). This is in accordance with the Tks transcriptome described in [33] with the only exception of oxidative phosphorylation pathway, responsible for production of energy in form of ATP, that in our dataset is the most abundant category.

We compared our data, obtained with a long read sequencing technology, with transcriptome sequences of Tks recently published [33], and observed that about 8% of our unigenes did not map on the reference transcriptome and hence those sequences were not represented in the published data. Besides, we observed that the matching sequences diverged of about 2% providing additional information on the genetic diversity in Tks which could be very useful for future studies. Moreover, we took advantage of the recently published draft genome structure of Tks [12] to extend the comparison of our dataset against the predicted genes of this genome. It resulted that more than 85% of unigenes mapped with a mean identity of about 98%. Noteworthy, most of the remaining 10,653 unigenes (15%) mapped on genomic regions not yet annotated as genes. Interestingly, 2566 of them were annotated as protein in at least one database; hence the corresponding genomic regions deserve to be further investigated for their putative gene content.

### Differential gene expression analysis

To describe the differentially expressed genes (DEGs) and identify genes putatively involved in NR biosynthesis, expression levels of assembled transcripts were calculated by RPKM method (Additional file 1: Table S7). Transcripts showing a differential abundance greater than 1.5-fold (*P*-value < 0.05) in at least one of HR vs. LR, HR vs. Y11 and LR vs. Y11 comparisons were assigned as DEGs (Additional file 1: Table S8).

The highest DEGs were in HR vs. Y11 comparison (3823) with 1849 up- and 1974 down-regulated genes; while lowest ones were in LR vs. Y11 comparison (3061), with 1422 up- and 1639 down-regulated. Finally, the HR vs. LR comparison showed 3084 DEGs, about equally distributed between up- and down-regulated.

DEGs data can be very useful to infer about the differential distribution of enzyme proteins in the metabolic machinery. Therefore, the KEGG Orthology-Based Annotation System (KOBAS) was used to perform KEGG pathway enrichment analyses (Additional file 1: Table S9).

The highest number of significantly enriched categories was observed in the genes upregulated in HR and LR vs. Y11 and, in particular, we observed a general downregulation of all the secondary metabolite biosynthetic pathways in Y11. In contrast, few categories were enriched for genes upregulated in Y11 vs. HR and LR, among them “fructose and mannose metabolism”, “oxidative phosphorylation” and “ribosome”.

Degradation of inulin, as a source of sugar via the glycolytic pathway, starts with its hydrolysis catalysed by 1-FEH. We found two *1-FEHs*, contig01048 and contig10015, upregulated in LR plants. Transcriptional data suggested a possible higher rate of sugar reutilization in LR with respect to HR plants. LR plants likely need higher amounts of sucrose as carbon source to sustain production of rubber competing metabolites.

“Sulfur metabolism” category was also enriched in HR vs. LR upregulated genes. Increased expression of the enzymes involved in assimilatory sulfate reduction (ATP sulfurylase 1, contig02141, ATP sulfurylase 2, contig01671, adenylyl-sulfate kinase, contig05025, and sulfite reductase 1, contig00659), may be related to an enhanced synthesis of glutathione that is necessary to maintain the redox homeostasis altered by the activity of oxidoreductases and by the high metabolic activity, in the roots and in the latex of HR plants. Supporting this hypothesis, Glutamate-cysteine ligase (contig01716), that is involved in the first step of glutathione biosynthesis, was upregulated in HR.

KEGG “peroxisome” category was enriched in the set of genes upregulated in HR vs. Y11. Peroxisomes are ubiquitous cell organelles that compartmentalize primarily oxidative metabolic reactions such as fatty acid beta-oxidation, the glyoxalate cycle, and photorespiration. Several peroxisome biogenesis genes were upregulated in HR vs. both Y11 and LR plants: peroxisome biogenesis protein 1 (contig02110), peroxisome biogenesis protein 12 (contig03525), peroxisome biogenesis protein 22-like (contig06219), and mitochondrial fission 1 protein A (contig10143). Moreover, contig04674, upregulated in HR, is a peroxisomal nicotinamide adenine dinucleotide carrier similar to *Arabidopsis At2g39970* that mediates the NAD+ import into peroxisomes [37], which is fundamental for lipid degradation (beta-oxidation). Hence, beta-oxidation may have a role in redox homeostasis and provides additional lipid derived acetyl-CoA that could be used for isoprenoid biosynthesis. Catalases that contribute to the antioxidant system in the peroxisomes, were highly expressed and modulated in our dataset: contig09705, 09080, 04121, 06273, 12349 were up- and contig15007, 04241, 10900 were down-regulated in HR vs. LR. Peroxisomes and genes involved in redox metabolism could thus be involved in the capacity to synthetize NR. The involvement of peroxisomes in isoprenoid biosynthesis in plants was proposed given that many MVA pathway enzymes can be localized to peroxisomes. Compartmentalization could provide a mechanistic explanation for channelling of FPP (farnesyl diphosphate) toward sterol or sesquiterpene biosynthesis [38]. Similarly, in Tks, peroxisomal compartmentalization of MVA pathway enzymes may promote the switching between production of NR and sesquiterpenes.

Genes included in the “biosynthesis of secondary metabolites” category were overrepresented among upregulated in LR vs. HR contigs. Among secondary metabolism enriched pathways, we found “stilbenoid, diarylheptanoid and gingerol biosynthesis”, containing genes involved in phenylpropanoid biosynthesis, and “limonene and pinene degradation” category that includes genes linked to sesquiterpene and monoterpene production. In both pathways cytochromes P450, belonging to the CYP71 clan, were highly represented and modulated. We hypothesize that secondary metabolites pathways may compete with the synthesis of NR: the up-regulation of these pathways, observed in LR plants, could negatively affect their ability to synthetize high amounts of rubber in the latex.

In order to validate the expression data obtained by RNA-seq experiments and to provide a better understanding of the molecular basis of the metabolic pathways involved, directly or indirectly, in the rubber biosynthesis, 27 genes were selected for qRT-PCR analysis and their relative abundance was compared in HR and LR plants. Genes involved in the MVA pathway, terpenoid and phenylpropanoid biosynthesis, as well as genes involved in response to oxidative stress and in transcription regulation have been included. Most of the selected genes (25 out of 27) presented very similar trends between qRT-PCR and RNA-seq data (Additional file 2: Figure S5A), as demonstrated by the high correlation coefficient (*R* = 0.726, *P* < 0.001) between the two approaches (Additional file 2: Figure S5B).

### Modulation of genes involved in rubber biosynthesis

The MVA pathway is the major source of IPP used in the formation of rubber chains. The rate limiting step of MVA pathway is the conversion of 3-hydroxy-3-methylglutaryl-coenzyme A to mevalonic acid, catalysed by HMGCR (3-hydroxy-3-methylglutaryl-coenzyme A reductase) [39, 40]. Plants have multiple-copy genes encoding for different HMGCRs, whose number varies from two in *Arabidopsis thaliana* up to twelve as recently observed in the Tks genome [12, 41].

We found 11 contigs presenting sequence homology with HMGCRs. Among them, 6 were partial sequences (05428, 13510, 13498, 08292, 08775 and 11010) and 5 were full length genes (01065, 00932, 02247, 01500 and 01645). The predicted protein sequences encoded by our 11 contigs and HMGCRs previously found in Tks and Tb, were aligned and included in a phylogenetic tree (Additional file 2: Figure S6 and S7).

**Fig. 3:**
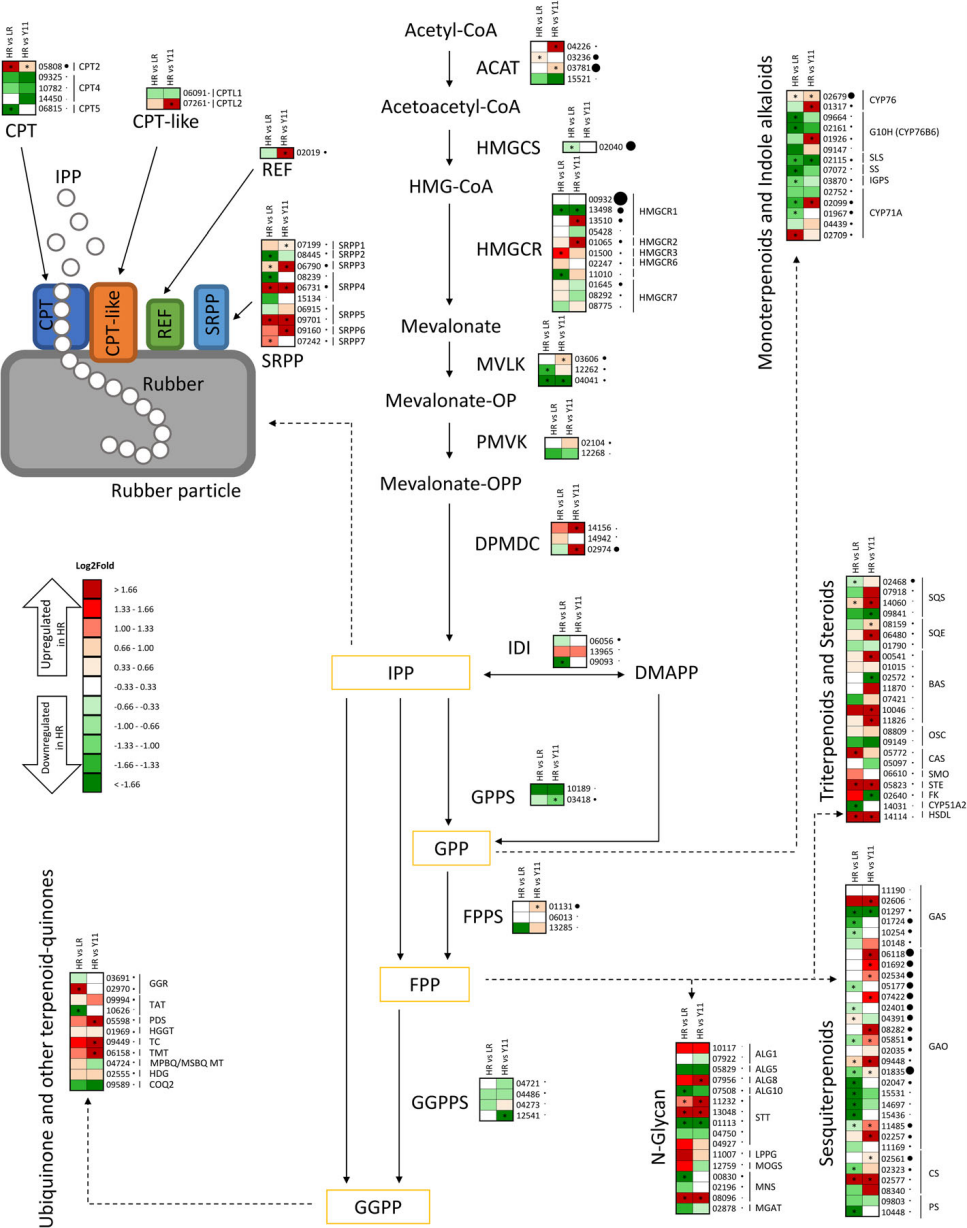
Expression of contigs involved in terpenoid biosynthesis in HR compared to LR and Y11 plants. On the top left corner, a schematic model of the rubber biosynthetic machinery. Relative levels of expression are showed by a color gradient from low (green) to high (red). Asterisks indicate significant differences (*P* < 0.05). Black dots sizes are proportional to the number of reads. Abbreviations: ACAT (Acetyl-CoA acetyltransferase), HMGCS (Hydroxymethylglutaryl-CoA synthase), HMGCR (Hydroxymethylglutaryl-CoA reductase), MVLK (Mevalonate kinase), PMVK (Phosphomevalonate kinase), DPMDC (Diphosphomevalonate decarboxylase), IPP (Isopentenyl pyrophosphate), IDI (Isopentenyl-diphosphate delta-isomerase), DMAPP (Dimethylallyl-diphosphate), GPP (Geranyl diphosphate), GPPS (Geranyl-diphosphate synthase), FPP (Farnesyl diphosphate), FPPS (Farnesyl diphosphate synthase), GGPP (Geranylgeranyl diphosphate), GGPPS (Geranylgeranyl diphosphate synthase), CPT (Cis-prenyl transferase), REF (Rubber elongation factor), SRPP (Small Rubber particle protein), GGR (Geranylgeranyl reductase), TAT (Tyrosine aminotransferase), PDS (4-hydroxyphenylpyruvate dioxygenase), HGGT (Homogentisate geranylgeranyltransferase), TC (Tocopherol cyclase), TMT (Tocopherol O-methyltransferase), MPBQ/MSBQ MT (2-methyl-6-phytyl-1,4-benzoquinone/2-methyl-6-solanyl-1,4-benzoquinone methyltransferase), HGD (Homogentisate 1,2-dioxygenase), COQ2 (4-hydroxybenzoate polyprenyltransferase), G10H (Geraniol 10-hydroxylase), SLS (Secologanin synthase), SS (Strictosidine synthase), IGPS (Indole-3-glycerol phosphate synthase), ALG1 (Asparagine-linked glycosylation protein 1), ALG5 (Asparagine-linked glycosylation protein 5), ALG8 (Asparagine-linked glycosylation protein 8), ALG10 (Asparagine-linked glycosylation protein 10), STT (Dolichyldiphosphooligosaccharide-protein glycosyltransferase), LPPG (Lipid phosphate phosphatase gamma), MOGS (Mannosyl-oligosaccharide glucosidase), MNS (Mannosyl-oligosaccharide 1,2-alpha-mannosidase), MGAT (Alpha-1,3-mannosyl-glycoprotein 2-beta-M-acetylglucosaminyltransferase), SQS (Squalene synthase), SQE (Squalene epoxidase), BAS (Beta-amirin synthase), OSC (Oxidosqualene cyclase), CAS (Cycloartenol synthase), SMO (C-4 Methyl sterol oxidase), STE (Delta(7)-sterol-C5-desaturase), FK (Delta(14)-sterol reductase, HSDL (Hydroxysteroid dehydrogenase-like), GAS (Germacrene A synthase), GAO (Germacrene A oxydase), CS (Costunolide synthase), PS (Parthenolide synthase)

Contig00932, homolog to a previously characterized TksHMGCR, AEA92686 [42], to utg17976.4 from Tks genome [12] and to HMGCR2 from Tb [5] (Additional file 2: Figure S7), was the most expressed member of HMGCR family, although not modulated (Fig. 3). This is in accordance with the expression levels reported for *utg17976.4* [12] and for *TbHMGCR2* [5]*.* Notably both are highly expressed in roots but not in latex suggesting a possible not prevalent role in this tissue and thus in NR biosynthesis.

**Fig. 4:**
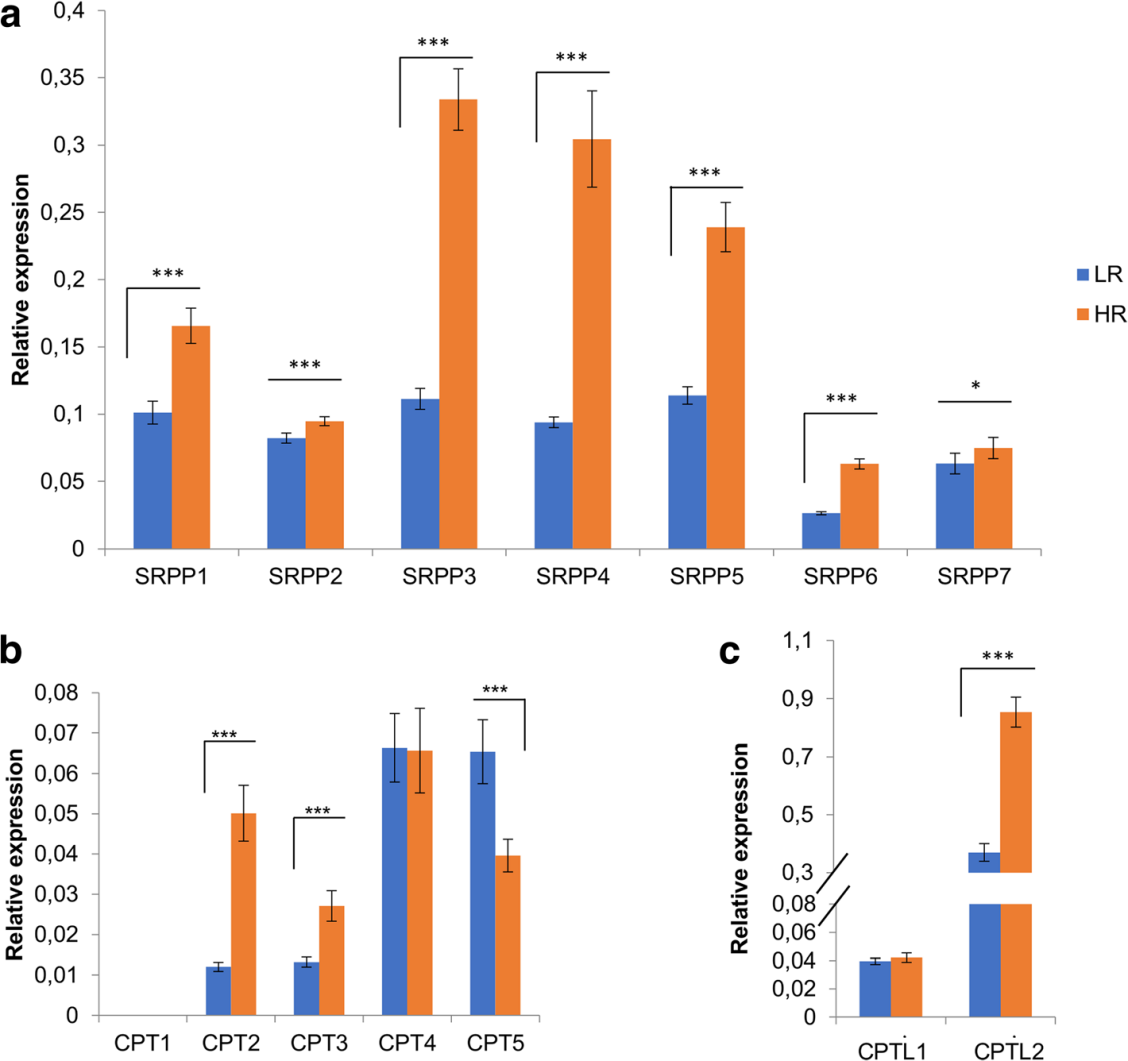
Relative expression of rubber related genes. Transcript levels of the analyzed genes were measured by qRT-PCR and were normalized to the expression of the housekeeping *GAPC2* gene. Asterisks indicate significant differences (*P < 0.05, **P < 0.01, ***P < 0.001; Student’s t-test). *SRPPs* (**a**). *CPTs* (**b**). *CPTLs* (**c**)

Contig01065 was significantly overexpressed in HR and LR vs. Y11 plants (Fig. 3) and it showed high sequence homology with utg10104.22 [12] and TbHMGCR1 [5] (Additional file 2: Figure S7). A role in rubber biosynthesis for TbHMGCR1, predominantly expressed in the latex, was previously proposed [5] and a similar expression pattern was observed also for its homolog in Tks [12]. Higher expression of contig01065 in HR vs. LR plants is evidenced by RNAseq and qRT-PCR (Fig. 4a).

Two contigs didn’t show homology with *HMGCR*s from the recently published Tks genome but may have a role in the synthesis of rubber precursors as suggested by their expression pattern: contig01500 that is significantly upregulated in HR vs. LR (Fig. 3 and Additional file 2: Figure S5A), and contig02247 that is more expressed in HR plants as confirmed by qRT-PCR (Additional file 2: Figure S5A). The latter showed a high degree of sequence similarity with TbHMGCR3 [5] (Additional file 2: Figure S7).

Among other genes involved in the MVA pathway, contig03236, coding for ACAT (Acetyl-CoA acetyl transferase), is the only upregulated in HR plants, while other contigs are not modulated or more expressed in LR plants (Fig. 3).

Sequential condensations of IPP to DMAPP (Dimethylallyl-diphosphate) lead to the synthesis of polyprenyl diphosphates of increasing size such as geranyl diphosphate (GPP, C10), farnesyl diphosphate (FPP, C15) or geranylgeranyl diphosphate (GGPP, C20), which could be further metabolized to monoterpenes, sesquiterpenes and diterpenes, respectively. In our experiments polyprenyl diphosphate synthases (*FPPS*, *GPPS* and *GGPPS*) showed a trend of higher expression in LR plants vs. HR (Fig. 3).

NR biosynthesis involves SRPP/REFs, CPTs and CPT-like proteins. In our dataset, 10 contigs were annotated as SRPPs (07199, 08445, 06790, 08239, 06731, 15134, 06915, 09701, 09160 and 07242), corresponding to 7 distinct SRPP proteins (SRPP1–7) (Fig. 3). Due to low expression level of several *SRPP* contigs inferred from transcriptomic data, their differential expression among HR and LR plants was further analysed by qRT-PCR. All *SRPP*s resulted more expressed in HR plants (Fig. 4a); in particular, *SRPP3*, *4*, and *5* were the most expressed and modulated, suggesting their involvement in the long chain cis-polyisoprene production in HR plants as already envisaged [12, 20]. Phylogenetic relationship with SRPPs/REFs from other rubber producing species (Additional file 2: Figure S8) showed that SRPP3, 4 and 5 can be grouped with genes from *Taraxacum* and Lettuce but not with genes from *Hevea*, confirming a possible divergence during evolution of gene functions and mechanisms for rubber particle stabilization between *Taraxacum* and *Hevea* [12]; REF proteins form a distinct phylogenetic group (Additional file 2: Figure S8). It is plausible to hypothesize a role in rubber biosynthesis also for *SRPP6* and *SRPP7*, upregulated in HR plants, although further experiments are needed to clarify their exact contribution. Interestingly, SRPP7 is part of a distinct phylogenetic group together with putative homolog genes from *Hevea*, Lettuce and *Taraxacum* (Additional file 2: Figure S8).

Five contigs (05808, 09325, 10782, 14450 and 06815) annotated as CPTs, were found in our analysis. Sequence similarity analyses and phylogenetic relationship with CPTs from other rubber producing species (Additional file 2: Figure S9) showed that Contig 05808 was homolog of TbCPT2 (JQ991925) [13] and that three contigs (09325, 10782, 14450) presented high similarity with LsCPT1 (KF752486). All these transcripts belong to the same phylogenetic group of HRT1 and HRT2, suggesting that this group is very important for NR biosynthesis, as already hypothesized in [12]. Contig06815 and its homolog LsCPT2 (KF752487) [15] are in a different phylogenetic group. In our transcriptome and in the assembled Tks genome a homolog of TbCPT1 was not found.

To analyse more in depth the expression of *CPT* genes in HR and LR plants, qRT-PCR experiments were performed (Fig. 4b). Expression of Tks homolog of *TbCPT1* was undetectable also by qRT-PCR confirming that this gene is probably absent in Tks. Contig05808 (*CPT2*) was significantly overexpressed in HR plants in both transcriptome and qRT-PCR analysis (Fig. 3 and Fig. 4b). *CPT4* (contig09325, 10782 and 14450), the *LsCPT1* homolog, was not significantly modulated, whilst contig06815 (*CPT5,* homolog of *LsCPT2*) was more expressed in LR plants (Fig. 3 and Fig. 4b). As a contig corresponding to *CPT3* could not be identified in our assembly, probably due to high sequence similarity with *CPT2*, we cloned and sequenced the homolog of *TbCPT3* (JQ991927) from R13 plant. *CPT3* expression was also evaluated by qRT-PCR and it was significantly upregulated in HR plants although less expressed than *CPT2*. We conclude that CPT2 and 3 probably have a major role in NR biosynthesis. It is plausible to hypothesize that the absence of CPT1 in Tks could give rise to a different rubber in terms of quality respect to Tb.

As reported in Hb and Tb, CPTs exhibit RTase activity when interact with the RTase activator (NgBR homolog) [11]. We identified contig06091, similar to *HbHRBP* and *LsCPTL1*, and contig07261 similar to *TbRTA* and *LsCPTL2* [11, 15] that are part of a distinct phylogenetic group (Additional file 2: Figure S9). Expression of contig06091 was not significantly modulated, whereas contig07261 was more expressed in HR plants as confirmed by qRT-PCR (Fig. 3 and Fig. 4c); this finding suggests an involvement of the latter in CPT RTase activity, as it occurs in lettuce and in Tb, where the silencing of *LsCPTL2* and *TbRTA* resulted in a dramatic decrease of NR in the latex [15, 19].

In contrast with recent literature [33], our analysis on the expression of the genes directly involved in rubber biosynthesis clearly showed that most of them, such as several *HMGCRs*, *CPTs, SRPPs* and *CPTLs*, were up regulated in high rubber producing plants. These patterns of expression are consistent with an improved capacity in producing NR, confirming a pivotal role of these genes in the biochemical pathways linked to the rubber. These differences can be explained by the different experimental design; in fact, in the present work we analysed plants differing in rubber production at a given data-point, whilst in [33] 9 months-old plants with a different total yield of rubber were compared. Thus, we can conclude that the rate of rubber production and consequently level of expression of NR biosynthetic genes is variable during time. A study of gene expression profiles in time series spanning the whole life cycle of the plants will help to fully elucidate the correlation between gene transcription and the rate of rubber synthesis, establishing when which gene is necessary.

### Transcriptional modulation of genes involved in putative NR competing pathways

Terpenoids are one of the most varied class of natural products with notorious biological functions and commercial applications. A subclass of terpenoids with known physiological and pharmaceutical importance is sesquiterpene lactones (STLs) [43]. Sesquiterpenoids are typically located in laticifers but they can also be found within the vacuoles of other cell types in the plant, specifically when produced in response to biotic stresses [21]. In *Taraxacum spp.*, major STLs, include taraxacolides, dihydro-lactucin, ixerin, taraxinic acids, and ainslioside [24].

We selected 31 contigs in our dataset putatively involved in STLs biosynthesis [44, 45]; interestingly, 14 of them were significantly overexpressed in LR vs. HR (Fig. 3), suggesting that in LR plants the excess of IPP, produced in the MVA pathway and not used in NR synthesis, may activate a higher rate of sesquiterpene synthesis.

Several contigs coding for enzymes involved in monoterpenoid and particularly in indole alkaloid biosynthesis were upregulated in LR vs. HR (Fig. 3), suggesting that also this class of compounds may transcriptionally compete with NR synthesis.

*Taraxacum* latex contains abundant quantities of pentacyclic triterpenes, including lupeol and amyrins, and other molecules that are more restricted to this genus, such as taraxasterol [9]. Squalene synthase and squalene epoxidase catalyse the first two steps in the pentacyclic triterpenes and sterols synthesis pathway, leading to the formation of 2,3-oxidosqualene. We found 4 squalene synthases, among them, contig02468 was significantly more expressed in LR plants (Fig. 3). Enzymes involved in phytosterols synthesis from 2,3-oxidosqualene, such as cycloarthenol synthase (contig05772) that catalyses the first biosynthetic step producing cycloartenol, delta7 sterol C-5 desaturase (contig05823) and hydroxysteroid dehydrogenase (contig14114), were significantly upregulated in HR vs. LR plants.

Phenolic acids and coumarins are abundant secondary compounds in dandelion roots [24]. Upstream steps in phenylpropanoid biosynthesis, including the shikimate pathway, the biosynthesis of aromatic aminoacids and their conversion to p-Coumaroyl-CoA showed a general higher expression in LR vs. HR plants (Additional file 2: Figure S10). Many genes involved in phenolic acids and coumarin biosynthesis (Hydroxycinnamoyltransferase/hydroxycinnamoyl-CoA:quinate hydroxycinnamoyltransferase, contig03203; p-coumarate 3-hydroxylase, contig02460; Caffeoyl shikimate esterase, contig05250, 04773, 08515; Caffeate O-methyltransferase, contig05261, 14800; Ferulate 5-hydroxylase, contig01965) were also significantly upregulated in LR vs. HR (Additional file 2, Figure S10). On the contrary, many genes involved in flavonoid biosynthesis were upregulated in HR vs. LR plants and even more in HR vs. Y11. CYP450s belonging to CYP80, 81 and 82 families showed a high expression in our dataset. These three families belong to the CYP71 clan that is involved in the modification of shikimate products and intermediates [46]. *CYP80s* were significantly upregulated in HR vs. LR, while *CYP82s* were downregulated (Additional file 2, Figure S10). This pattern of expression probably reflects the differential synthesis of secondary compounds in HR and LR plants. We infer that in LR plants is advisable a higher synthesis of phenolic acids and derived compounds such as coumarins whereas the synthesis of flavonoids is more active in HR plants.

As shown in Additional file 2: Figure S10, another class of highly expressed and modulated transcripts, the root allergen proteins, were mostly upregulated in LR vs. HR. This class of proteins is abundant in *Taraxacum officinale* roots and their function is considered related to pathogenesis [47].

An overall competition among synthesis of different classes of specialised metabolites in Tks is evidenced at transcriptional level. In the latex, isoprenoid synthesis likely switches between the production of NR and phytosterols in HR plants and of sesquiterpene lactones and monoterpenoids in LR plants, evidencing a competition in the use of the IPP as precursor. In fact, a reduced NR synthesis due to *CPT* silencing in Tb plants, caused an increase of pentacyclic triterpenes and sterols [9]. The control of the synthesis of different specialised metabolites can be exerted with numerous mechanisms. As reported above, genes involved in peroxisome biogenesis are modulated and peroxisomes may have a role in determination of MVA flux toward synthesis of different terpenoid classes [38].

The AP2-ERF and WRKY transcription factor families as well as MYC2 and the hormone methyl jasmonate are quickly emerging as important regulators of terpene biosynthesis [48–51]. Four contigs belonging to AP2-ERF family (04535, 09007, 07313 and 07055) were significantly modulated: the first three were more expressed in HR plants, the last in LR plants. Contigs 05164, 10242, 02598, 10367 and 07938, annotated as WRKY family members, were also modulated, with the first two up- and the others down-regulated in HR vs. LR plants. Moreover, contig 05164 shares similarity with *HbWRKY1* implicated in the regulation of latex production and strongly induced by abscisic acid, ethylene and jasmonate [52]. Two contigs, 00569 and 07332, annotated as MYC2 and MYC2-like, were significantly upregulated in HR plants suggesting a possible role in the regulation of NR synthesis.

## Conclusions

Plants need to defend themselves against diverse and dynamic herbivore communities, thus variability in the synthesis of different defence metabolites greatly increases their fitness. Latex is a mixture of secondary metabolites, enzymes and stress/defense proteins which play a key role in defense against pathogens, parasites and herbivores [53, 54]. In this work we identified two groups of plants (HR and LR) with divergent activity in the synthesis of NR, a specific latex metabolite. Beside NR synthesis genes, in HR roots, a number of transcripts related to phytosterols and flavonoids production appears to be upregulated with respect to LR plants. On the other hand, many genes linked to the biosynthesis of sesquiterpene lactones, monoterpenes, phenylpropanoids such as phenolic acids and coumarins and root allergen proteins, are more expressed in LR roots compared to HR ones. It is plausible to speculate about a possible divergence in the overall production of root metabolites and consequently of latex composition between HR and LR plants. Moreover, in Y11, the almost absence of latex is correlated to the downregulation of all the secondary metabolite biosynthetic pathways and to the upregulation of several genes involved in the primary metabolism, compared to both HR and LR plants. We hypothesize that Y11 is mainly working in the production of energy and reserves at the expense of defense compounds. This is also in accordance with previous observations of competition between inulin and NR synthesis in Tks [23]. In our view, these findings provide useful information regarding NR production and the complex relationships among the different biochemical pathways involved in Tks root physiology.

## Methods

### Plant material

Seeds from nine wild Tks populations were obtained from USDA-ARS (Regional Plant Introduction Station, Washington State University, USA) with the following accession numbers W6 35156, W6 35164, W6 35165, W6 35166, W6 35168, W6 35170, W6 35172, W6 35173, W6 35176. About 20 seeds/population were germinated on Petri dishes with 10% agar in H_2_O at the beginning of March 2014. Within two weeks, germinated plantlets were first transferred to polystyrene trays (20 ml holes filled with standard commercial cultivation substrate) and after two months (early in May) were transplanted to 2.5 l polyethylene pots filled with 50% cultivation substrate and 50% sifted, medium textured field soil. Potted plants were cultivated in protected environment from May to November (tunnel covered by an anti-insect net), under near natural condition for photoperiod, temperature, humidity and exposure to atmospheric events, in ENEA Trisaia Research Center (40°09′47.4″N 16°38′00.1″E, Basilicata region, Italy) and shaded during summer, irrigated, fertilized and treated against pests and fungal pathogens. Weeds were manually removed, and pots were randomly moved every two weeks to minimize position effects. Plants were sampled late in November when they were about 9 months old.

### Rubber analysis

Rubber analysis was performed in November 2014 when plants were expected to be active in rubber synthesis and accumulation. Quantification was done as described by Cornish, K. and colleagues [55] with some modification. Briefly plants were removed from pots and cleaned with water. Latex was harvested from excised roots in four replicates, collected in 0.5 ml Eppendorf tubes, and quickly weighed. Immediately after, 300 μl of ice cold extraction buffer (0.1% Na_2_SO_3_ adjusted to pH 11 with NH_4_OH) was added and the mixture was centrifuged at 10,000 rcf for 15′. Centrifugation fractionated latex samples into three phases, from top to down: the rubber, aqueous and pellet phase. Glacial acetic acid (50 μl) was gently pipetted on the upper phase to promote the coagulation of the rubber and the tubes were centrifuged at 10,000 rcf for 15′ again. The upper layer made of coagulated rubber was removed, rinsed in deionized water, placed on filter paper and dried overnight at 37 °C before determination of its weight. Low variation among replicates was observed showing that latex harvested from different root branches and in different positions is homogeneous as per rubber content.

### RNA preparation, cDNA library construction, and sequencing

Root tissues from three HR plants (R13, F3 and O7) from three LR plants (Y1, V16 and Z2) and from Y11 plant were collected and stored at − 80 °C. Total RNAs were extracted using innuPREP Plant RNA Kit (Analytik Jena, Germany) according to the manufacturer’s protocol. RNA quality and concentration were estimated by RNA 6000 Nano chip on Bioanalyzer 2100 system (Agilent Technologies, USA) and by Nanodrop™ 1000 Spectrophotometer (Thermo Fisher Scientific, USA), respectively. Equimolar amount of RNAs from HR plants and from LR plants were pooled to eliminate the variation between individual plants and to obtain representative samples. One microgram of RNA per sample (HR, LR and Y11) was used for cDNAs synthesis using SMART PCR cDNA Synthesis kit (Clontech, USA) following manufacturer’s protocol. Double stranded cDNAs were purified using QIAquick PCR purification kit (Qiagen, Germany) and quantified by Quant-iT™PicoGreen-Assay Kit on NanoDrop™ 3300 Fluorospectrometer (Thermo Fisher Scientific, USA). Three shotgun libraries were obtained using GS Rapid Library prep kit (Roche Diagnostics Corporation, Switzerland) according to manufacturer’s protocol and then sequenced using a 454 GS FLX+ Titanium Sequencer (Roche Diagnostics Corporation, Switzerland) following manufacturer’s protocol.

### Quality control, assembly, and functional annotation

The raw 454 reads were trimmed by removing of adaptor sequences, primers, poly A/T stretches and short reads using customized SeqClean (https://sourceforge.net/projects/seqclean/files/) software. High quality reads were assembled into unique putative transcripts, termed as unigenes (including contigs and singletons), using the GS De Novo Assembler Software v2.9 (Roche Diagnostics Corporation, Switzerland), with default parameters setting.

All unigenes were annotated, using stand-alone NCBI Blast program with an E-value threshold of 1e^- 5^, against nr (NCBI non-redundant protein sequences; http://www.ncbi.nlm.nih.gov/), TAIR (The Arabidopsis Information Resource; https://www.arabidopsis.org/) and UniProtKB/SwissProt (a manually annotated and reviewed protein sequence; http://www.uniprot.org/) databases. Annotation of contigs cited in the text and in the figures was manually revised.

Functional annotation and GO classification of unigenes were carried out by Blast2GO PRO software (https://www.blast2go.com/blast2go-pro), with default settings. Briefly, Blast2GO mapping was used to perform the GO annotation based on the blast results (nr database, E-value <1e^− 5^). KEGG (Kyoto Encyclopedia of Genes and Genomes) annotation and classification (E-value <1e^- 5^) were performed using Kobas 3.0 software (KEGG Orthology-Based Annotation System). Mapping comparisons against the Tks genome and reference transcriptome were carried out using NCBI Magic BLAST 1.3.0 (https://ncbi.github.io/magicblast/) with default parameters.

### Differential gene expression analysis

The normalized gene expression levels were calculated as described in [56] and were reported as reads per kilobase per million reads mapped (RPKM). For each pairwise comparison (HR vs. LR, HR vs. Y11 and LR vs. Y11) we calculated the fold change (FC) as a ratio between RPKM values. Significant differentially-expressed transcripts were selected according to their relative read abundances into the three libraries, as decribed by Stekel and Falciani (R test with a significance threshold of 0.05) using IDEG6 web tool [57, 58]. Transcripts with a *P*-value < 0.05 and a FC ≥ 1.5 were assigned as differentially expressed (DEGs).

Kobas 3.0 software (http://kobas.cbi.pku.edu.cn/) was used to correlate DEGs to metabolic pathways. Significantly enriched pathways were identified applying hypergeometric distribution / Fisher’s exact test (*p* ≤ 0.05) [35].

### Quantitative RT-PCR

cDNA was synthesized from 1 μg of RNA with oligo(dT)_16_ using SuperScript® IV kit (Invitrogen/ Thermo Fisher Scientific, USA) according to manufacturer’s instructions. qRT-PCR was performed using an ABI Prism® 7900HT instrument (Applied Biosystems/Thermo Fisher Scientific, USA) and Platinum® SYBRGreen® qPCR SuperMix-UDG with ROX (Invitrogen/Thermo Fisher Scientific, USA) according to manufacturer’s instructions. PCR conditions were: 5′ at 95 °C followed by 45 cycles at 95 °C for 15″ and at 58 °C for 60″. Quantification was performed using standard dilution curves for each studied gene fragment and the data were normalized for the quantity of housekeeping *GAPC2* (Glyceraldehyde-3-phosphate dehydrogenase C2) and *EF2* (Elongation factor 2) transcripts, which were both highly expressed and not modulated according to RNAseq data. Primers were designed using Primer Express Software (Applied Biosystems/Thermo Fisher Scientific, USA), except those for the amplification of *CPT1*, *CPT2* and *CPT3* which were manually designed. Primer pairs were validated with Amplify Software (University of Wisconsin, USA) and by BLAST analysis in order to avoid amplification of possible off-target sequences. Sequences of the selected primers are listed in Additional file 1: Table S10 (genes for HR vs. LR data validation), S11 (SRPP and SRPP-like genes), S12 (CPT and CPT-like genes) and S13 (housekeeping genes).

### CPT3 cloning

*CPT3* was amplified from genomic DNA of R13 plant using primer CPT3fw 5′ ATG CAA GTG AAT CCA ATC 3′ and CPT3rev 5′ TTA TGC CTG CTT CTT CTT C 3′ designed on TbCPT3 sequence, cloned using TA Cloning® Kit with pCR™2.1 (Invitrogen/ Thermo Fisher Scientific, USA) in TOP10 chemically competent cells and sequenced. Intron sequence was identified by comparison with TbCPT3 transcript sequence.

### Protein sequences analysis

Protein sequences encoded by selected contigs from our dataset and *CPT3* were obtained using the Expasy Translate Tool (http://web.expasy.org/translate/). Sequence alignments and phylogenetic trees were made with Clustal Omega [59] analysis within CLC Genomics Workbench v9.5.2 software (Qiagen, Germany).

## Additional files

Additional file 1: **Table S1.** Analysis of rubber content. **Table S2.** Summary of sequencing results from HR, LR and Y11 libraries. **Table S3.** De Novo assembly results. **Table S4.** Summary of functional annotation of Tks transcripts by BLAST search against public databases. **Table S5.** Detailed annotation of Tks transcripts by BLAST search against public databases. **Table S6.** KEGG pathway assignment. **Table S7.** Expression level of assembled contigs. **Table S8.** Differentially expressed genes (DEGs). **Table S9.** KEGG Enrichment analysis. **Table S10.** qRT-PCR primers for selected genes for HR/LR data validation. **Table S11.** qRT-PCR primers for *SRPP* genes. **Table S12.** qRT-PCR primers for *CPT* and *CPTL* genes. **Table S13.** qRT-PCR primers for housekeeping genes. (XLSX 9447 kb)

Additional file 2: **Figure S1.** Average rubber content (% *w*/w) of the accessions analyzed. **Figure S2.** The gene ontology (GO) distributions of the unigenes. **Figure S3.** Enzyme Code distributions (EC) of the unigenes for the six main classes of enzymes. **Figure S4.** KEGG pathway classification map. **Figure S5.** Comparison of HR/LR ratios (expressed as log2) produced with RNA-Seq and qRT-PCR of 27 selected genes. **Figure S6.** Sequence alignment of contigs presenting sequence homology with HMGCRs. **Figure S7.** Phylogenetic relationship of HMGRs. **Figure S8.** Phylogenetic relationship of SRPPs/REFs. **Figure S9.** Phylogenetic relationship of CPTs/CPTLs. **Figure S10.** Expression of contigs involved in phenylpropanoid and flavonoid biosynthesis, and belonging to CYP80–81-82 and RAP (Root allergen protein) categories in HR compared to LR and Y11 plants. (PDF 1638 kb)

## Abbreviations

1-FEH: 1-Fructan exohydrolase
ACAT: Acetyl-CoA acetyl transferase
CPT: Cis-prenyltransferase
DEG: Differentially expressed gene
DMAPP: Dimethylallyl-diphosphate
EC: Enzyme commission
EF2: Elongation factor 2
FPP: Farnesyl diphosphate
FPPS: Farnesyl diphosphate synthase
GAPC2: Glyceraldehyde-3-phosphate dehydrogenase C2
GGPP: Geranylgeranyl diphosphate
GGPPS: Geranylgeranyl diphosphate synthase
GO: Gene ontology
GPP: Geranyl diphosphate
GPPS: Geranyl diphosphate synthase
Hb: *Hevea brasiliensis*
HMGCR: 3-hydroxy-3-methylglutaryl-coenzyme A reductase
HR: High rubber
HSD: Honest significant difference
IPP: Isopentenyl pyrophosphate
KEGG: Kyoto Encyclopedia of Genes and Genomes
KOBAS: KEGG Orthology-Based Annotation System
LR: Low rubber
LSM: Least Square Mean
MVA: Mevalonic acid
NgBR: Nogo-B receptor
NR: Natural rubber
nr: non redundant
qRT-PCR: quantitative reverse transcripase polymerase chain reaction
REF: Rubber elongation factor
RP: Rubber particle
RPKM: Reads per kilobase per million
RTase: Rubber transferase
SD: Standard deviation
SRPP: Small rubber particle protein
STL: Sesquiterpene lactone
TAIR: The Arabidopsis information resource
Tb: *Taraxacum brevicorniculatum*
Tks: *Taraxacum kok-saghyz*
