# Supplementary material for: Comparative transcriptomics between high and low rubber producing Taraxacum kok-saghyz R. plants
Source: BMC Genomics. 2018 Dec 4;19:875. doi: 10.1186/s12864-018-5287-4 (PMC6280347; doi:10.1186/s12864-018-5287-4)
Supplement: Supplementary file 2 — Figure S1. Average rubber content (% w/w) of the accessions analyzed. Figure S2. The gene ontology (GO) distributions of the unigenes. Figure S3. Enzyme Code distributions (EC) of the unigenes for the six main classes of enzymes. Figure S4. KEGG pathway classification map. Figure S5. Comparison of HR/LR ratios (expressed as log2) produced with RNA-Seq and qRT-PCR of 27 selected genes. Figure S6. Sequence alignment of contigs presenting sequence homology with HMGCRs. Figure S7. Phylogenetic relationship of HMGRs. Figure S8. Phylogenetic relationship of SRPPs/REFs. Figure S9. Phylogenetic relationship of CPTs/CPTLs. Figure S10. Expression of contigs involved in phenylpropanoid and flavonoid biosynthesis, and belonging to CYP80–81-82 and RAP (Root allergen protein) categories in HR compared to LR and Y11 plants. (PDF 1638 kb) [file 12864_2018_5287_MOESM2_ESM.pdf]

Original Research Article

Comparative transcriptomics between high and low rubber producing Taraxacum kok-saghyz R. plants.

Francesco Panara[§], Loredana Lopez[§], Loretta Daddiego[§], Elio Fantini, Paolo Facella[*] & Gaetano Perrotta

ENEA, Italian National Agency for New Technologies Energy and Sustainable Economic Development, Trisaia Research Center, 75026 Rotondella (MT), Italy

[§]These authors contributed equally to this work

Correspondence and requests for materials should be addressed to P.F. (email: paolo.facella@enea.it)

Supplementary Figures

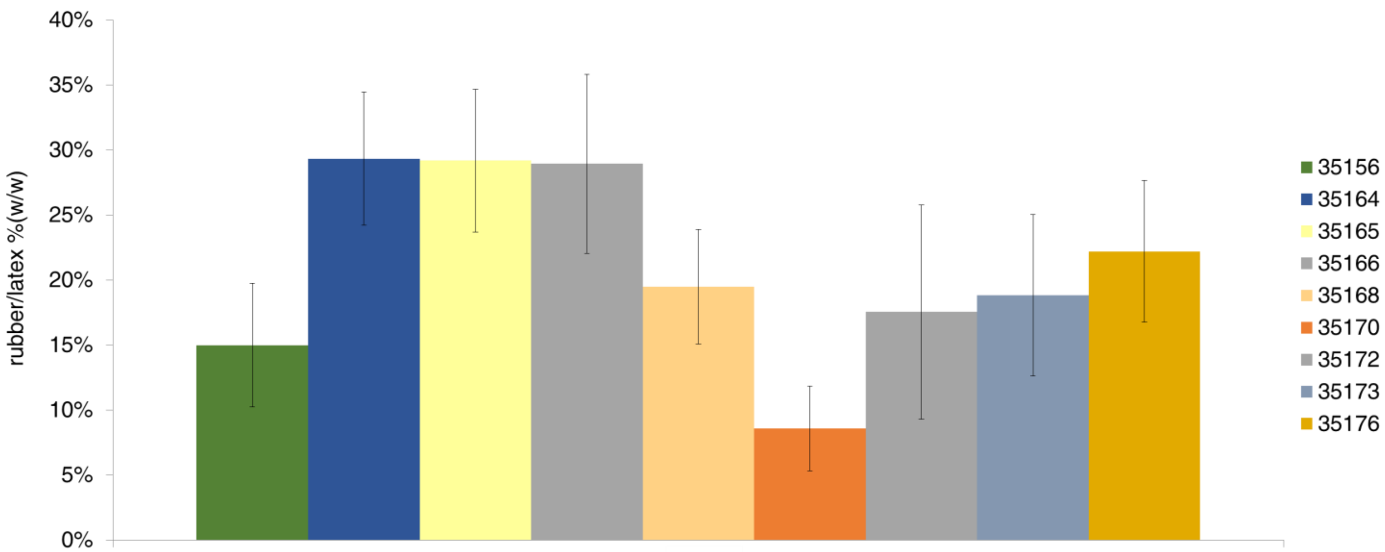

Figure S1. Average rubber content (% w/w) of the accessions analyzed in this paper. Error bars represent standard deviations.

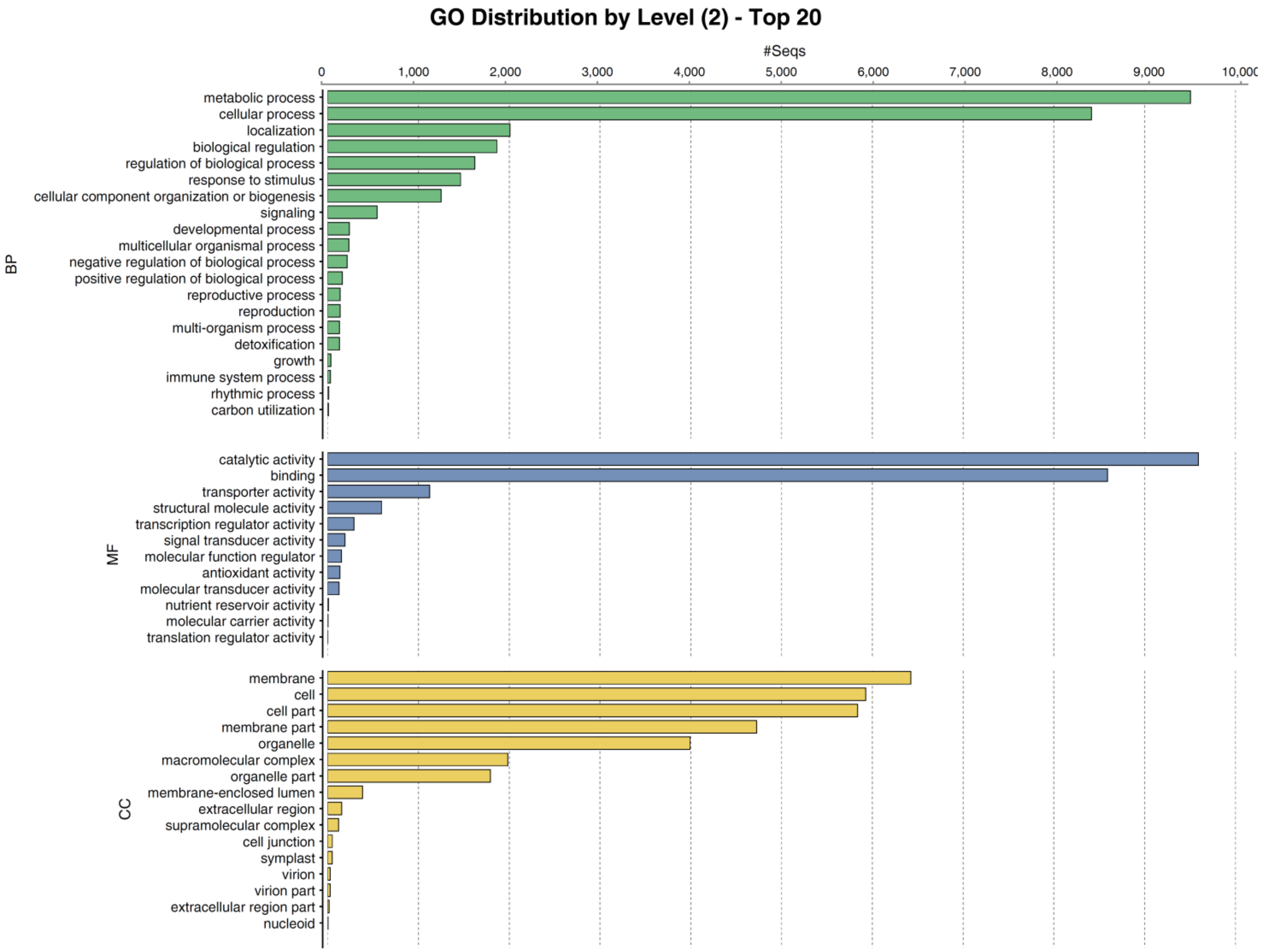

Figure S2. The gene ontology (GO) distributions of the unigenes for Molecular function (MF) Biological Process (BP) and Cellular Component (CC) categories.

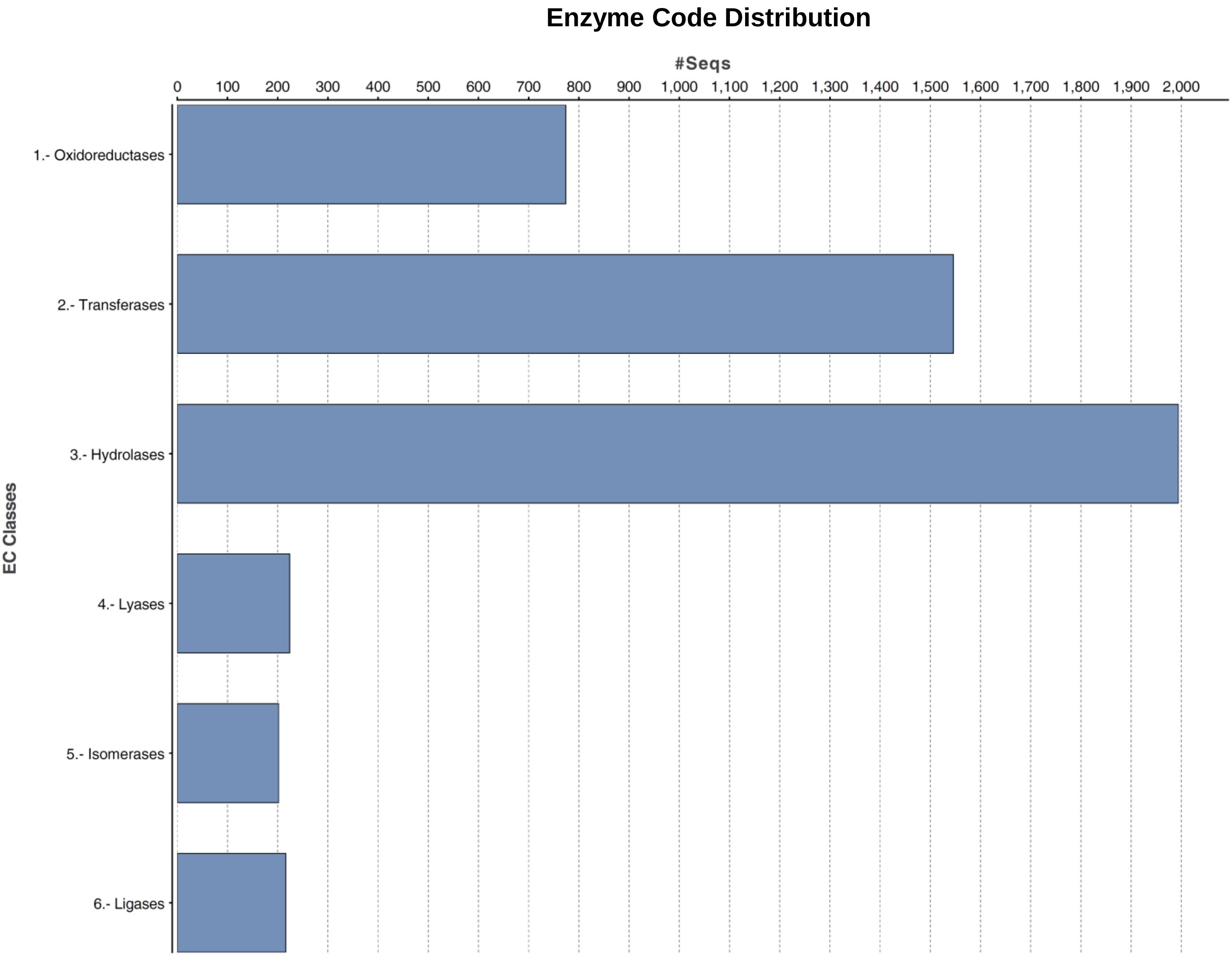

Figure S3. Enzyme Code distributions (EC) of the unigenes for the six main classes of enzymes.

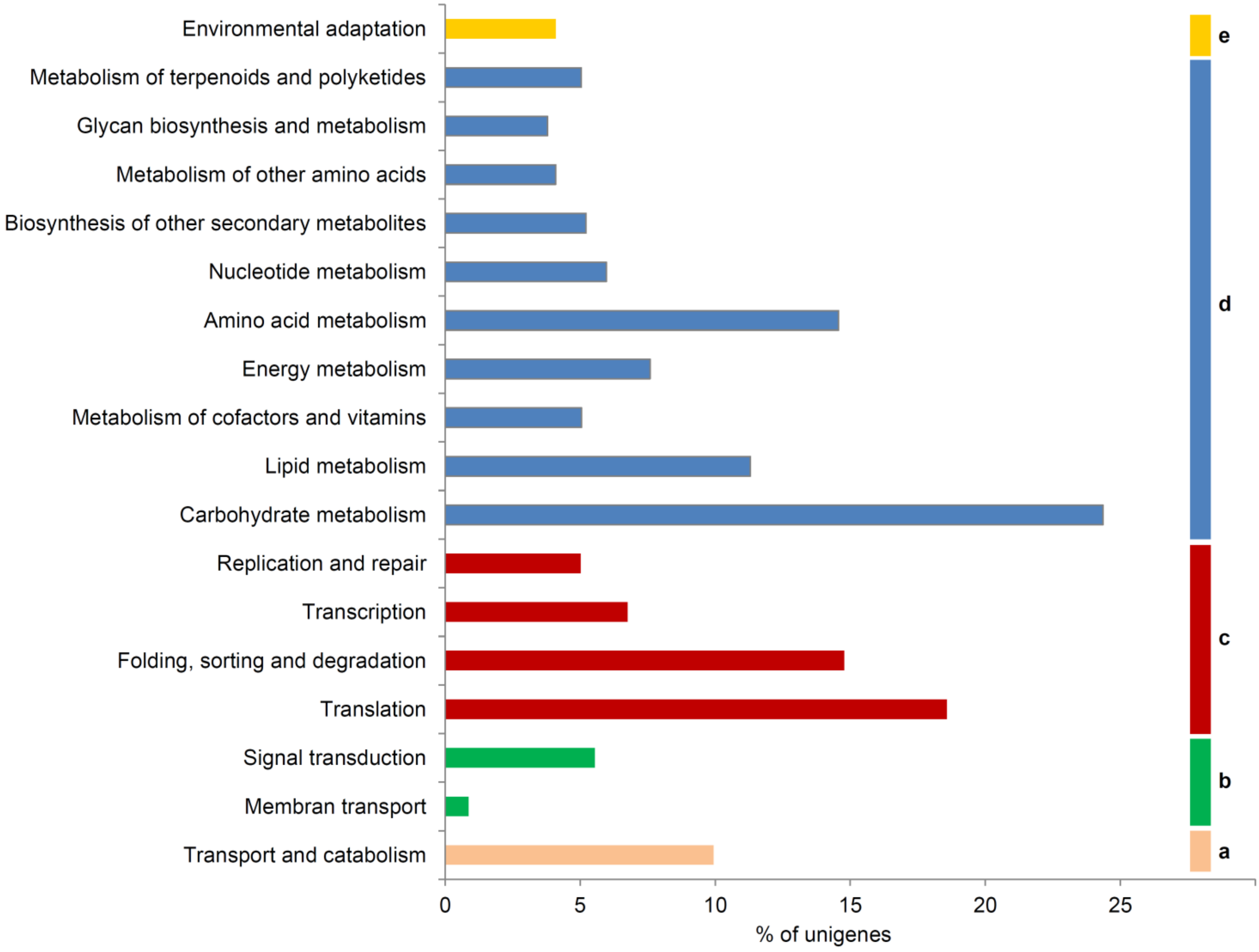

Figure S4. KEGG pathway classification map. Genes were grouped according to the biological pathway: Cellular Processes (a); Environmental Information Processing (b); Genetic Information Processing (c); Metabolism (d); Organismal Systems (e).

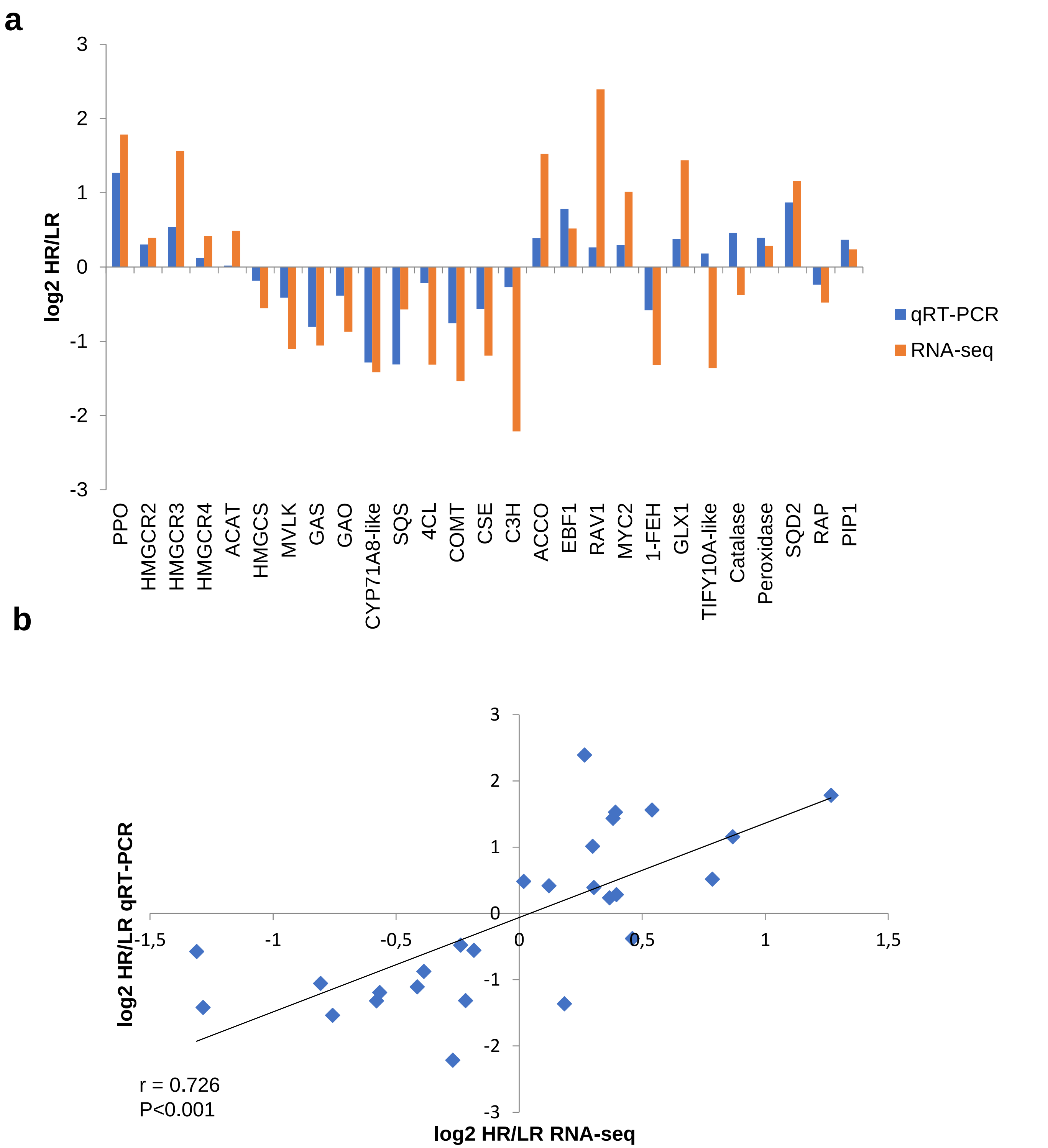

Figure S5. Comparison of HR/LR ratios (expressed as log2) produced with RNA-Seq and qRT-PCR of 27 selected genes. The ratios are between transcript levels of library HR and library LR, which were expressed as RPKM in the RNA-Seq experiment and as relative expression values (after normalization to the housekeeping genes GAPC2 and EF2) in the qRT-PCR experiment (**a**). Scatter plot correlation between qRT-PCR and RNA-Seq log2 HR/LR ratios (Pearson's correlation coefficient = 0.726, significance of the slope of the regression line: $p < 0.001$, Student's t-test) (**b**). Abbreviations: PPO (Polyphenol oxidase), HMGCR (Hydroxymethylglutaryl-CoA reductase), ACAT (Acetyl-CoA acetyltransferase), HMGCS (Hydroxymethylglutaryl-CoA synthase), MVLK (Mevalonate kinase), GAS (Germacrene A synthase), GAO (Germacrene A oxydase), SQS (Squalene synthase), 4CL (4-Coumarate-CoA Ligase), COMT (Caffeate O-methyltransferase), CSE (Caffeoylshikimate esterase), C3H (4-Coumaric acid 3'-hydroxylase), ACCO (Aminocyclopropanecarboxylate oxidase), EBF1 (EIN3-binding F-box protein 1), RAV1 (Ralated to ABI3/VP1 1), 1-FEH (1-Fructan exohydrolase), GLX1 (Glyoxalase 1), SQD2 (Sulfoquinovosyl transferase 2), RAP (Root allergen protein), PIP1 (Plasma membrane intrinsic protein 1).

HMGCR

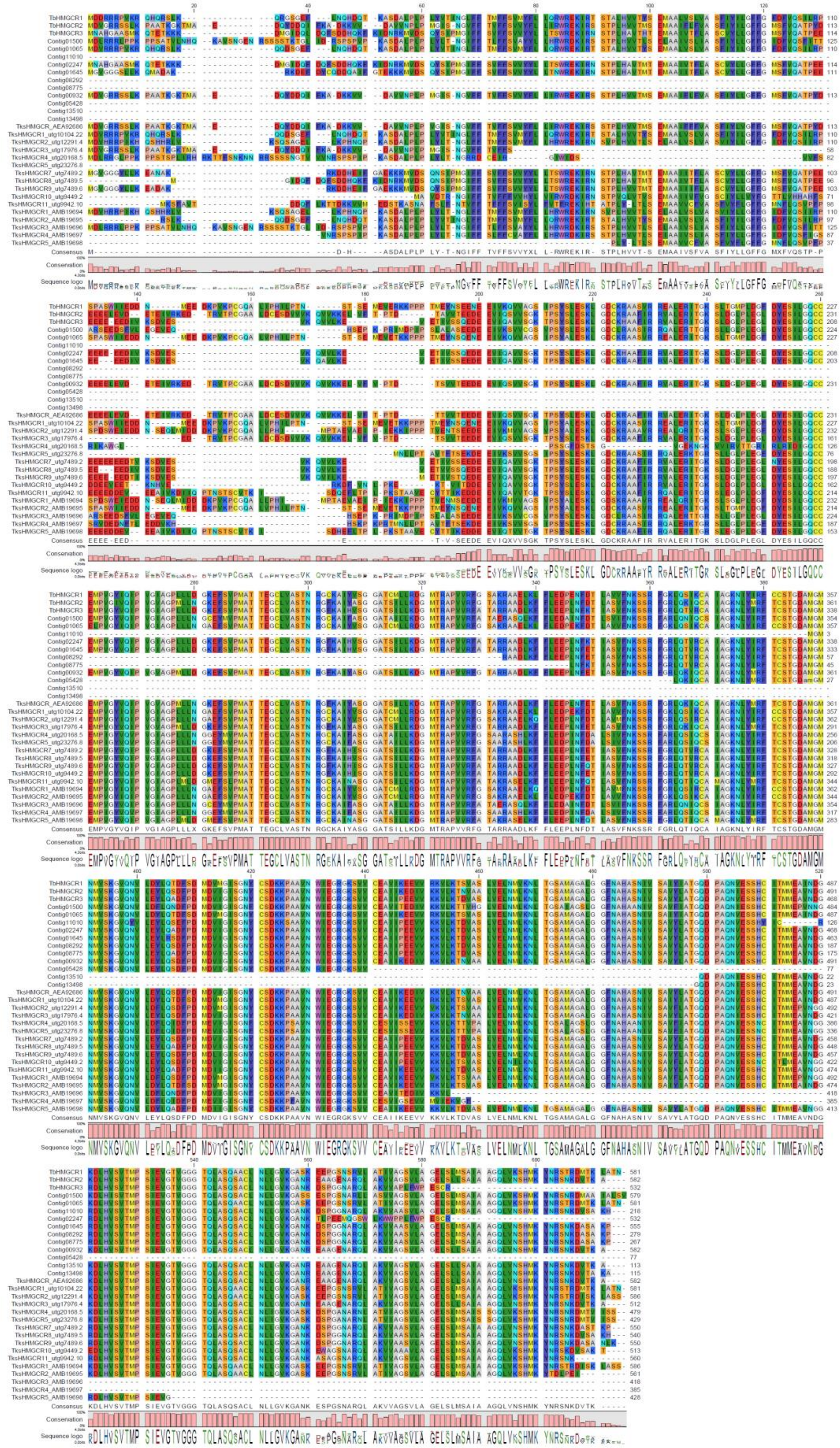

Figure S6. Sequence alignment of contigs presenting sequence homology with HMGCRs. Tb, Taraxacum brevicorniculatum (TbHMGCR1-3, from Van Deenen et al., 2012); Tks, Taraxacum kok-saghyz (TksHMGCR, AEA92686 from Ponciano et al., 2014, TksHMGCR1-5, AMB19694-8 from GenBank and other TksHMGCRs from Lin et al., 2017).

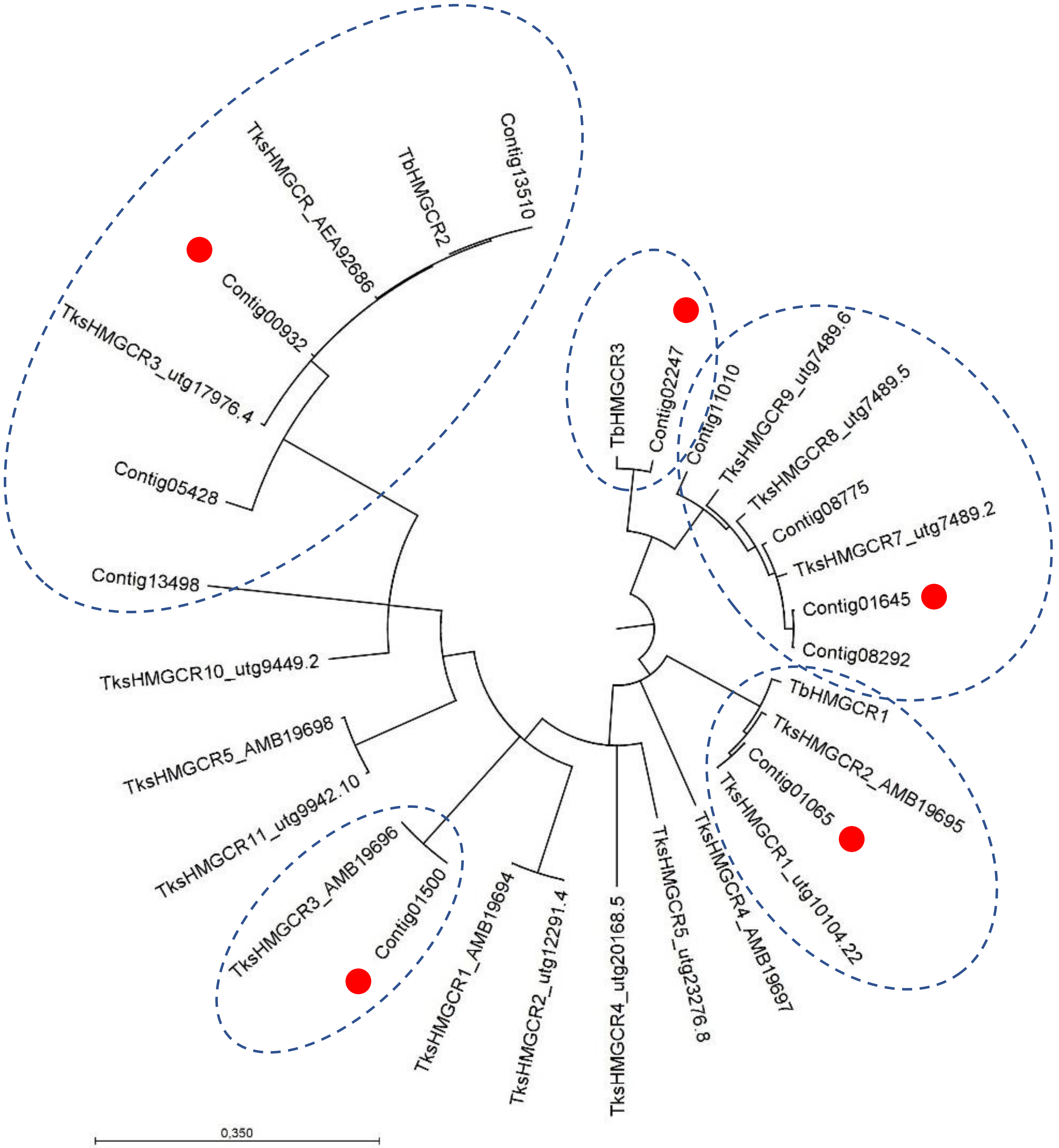

Figure S7. Phylogenetic relationship of HMGRs. Tb, Taraxacum brevicorniculatum (TbHMGCR1-3, from [5]); Tks, Taraxacum kok-saghyz (TksHMGCR, AEA92686 from [42], TksHMGCR1-5, AMB19694-8 from GenBank and other TksHMGCRs from [12]). Red dot indicates contigs coding for full length proteins.

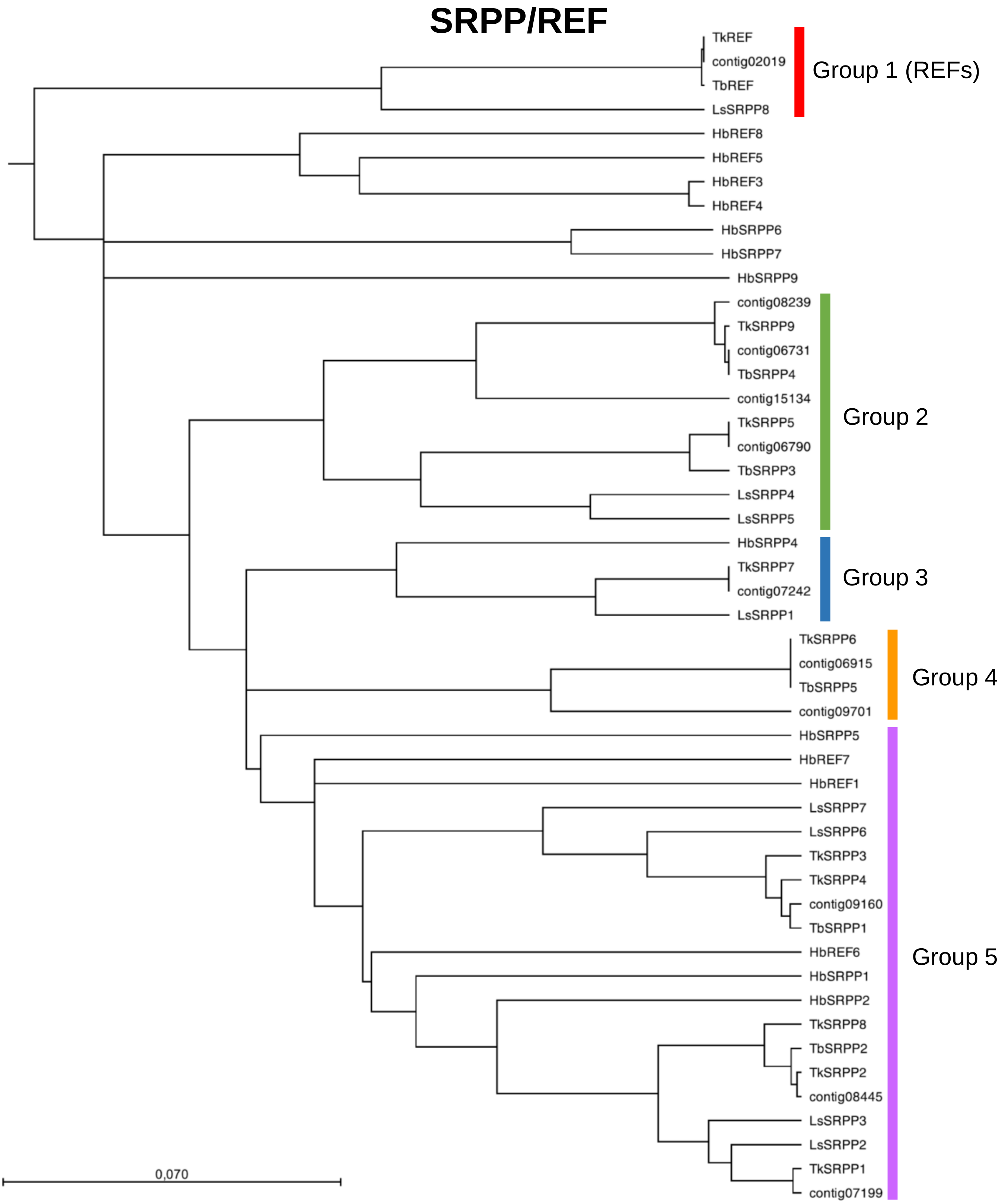

Figure S8. Phylogenetic relationship of SRPPs/REFs. Tk, Taraxacum kok-saghyz (TkSRPP1-9, TkREF from [12]). Tb, Taraxacum brevicorniculatum (TbSRPP1-5 from [18] and TbREF from [17]); Hb, Hevea brasiliensis (HbREFs and HbSRPPs from Tang et al.,2016 Nature Plants, 2:16073), Ls, Lactuca sativa (LsSRPPs from Reyes-Chin-Wo et al., 2017 Nature Communications, 8:14953). Contig02019 = REF. Contig07199 = SRPP1, contig08445 = SRPP2, contig06790 = SRPP3, contig08239, 06731, 15134 = SRPP4, contig06915 and 09701 = SRPP5, contig09160 = SRPP6 and contig07242 = SRPP7. Groups harbouring Tks genes are indicated by a coloured bar.

CPT/CPTL

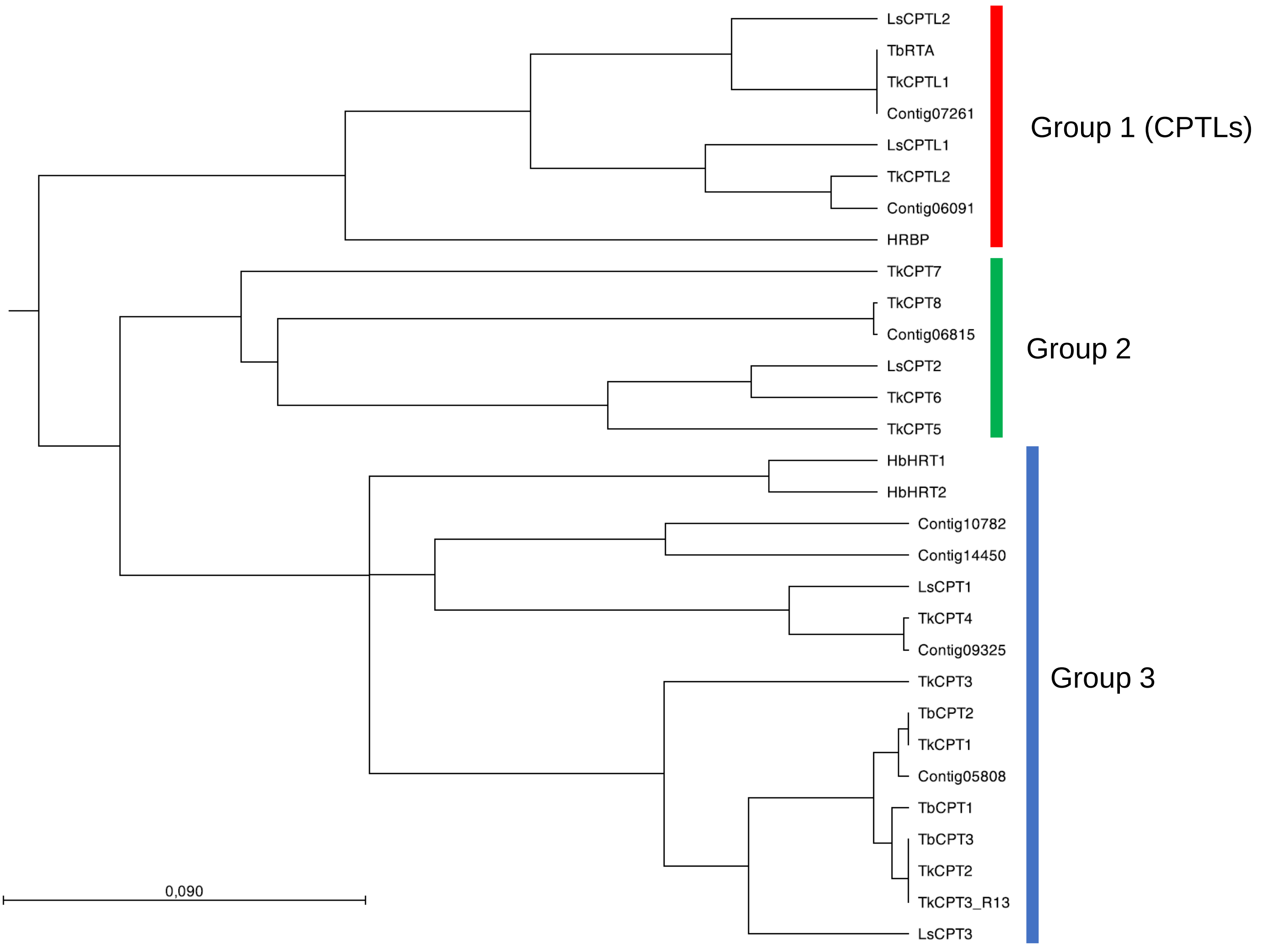

Figure S9. Phylogenetic relationship of CPTs/CPTLs. Tk, Taraxacum kok-saghyz (TkCPT1-8, TkCPTL1-2 from [12]). Tb, Taraxacum brevicorniculatum (TbCPT1-3 from [13] and TbRTA from [19]); Hb, Hevea brasiliensis (HbHRT1-2 and HRBP from Tang et al.,2016 Nature Plants, 2:16073), Ls, Lactuca sativa (LsCPT1-3 and LsCPTL1-2 from [15]). Contig05808=CPT2, contig09325, 10782 and 14450=CPT4, contig06815=CPT5. TkCPT3_R13 is the TbCPT3 homolog cloned from plant R13. The three main groups are indicated by a coloured bar.

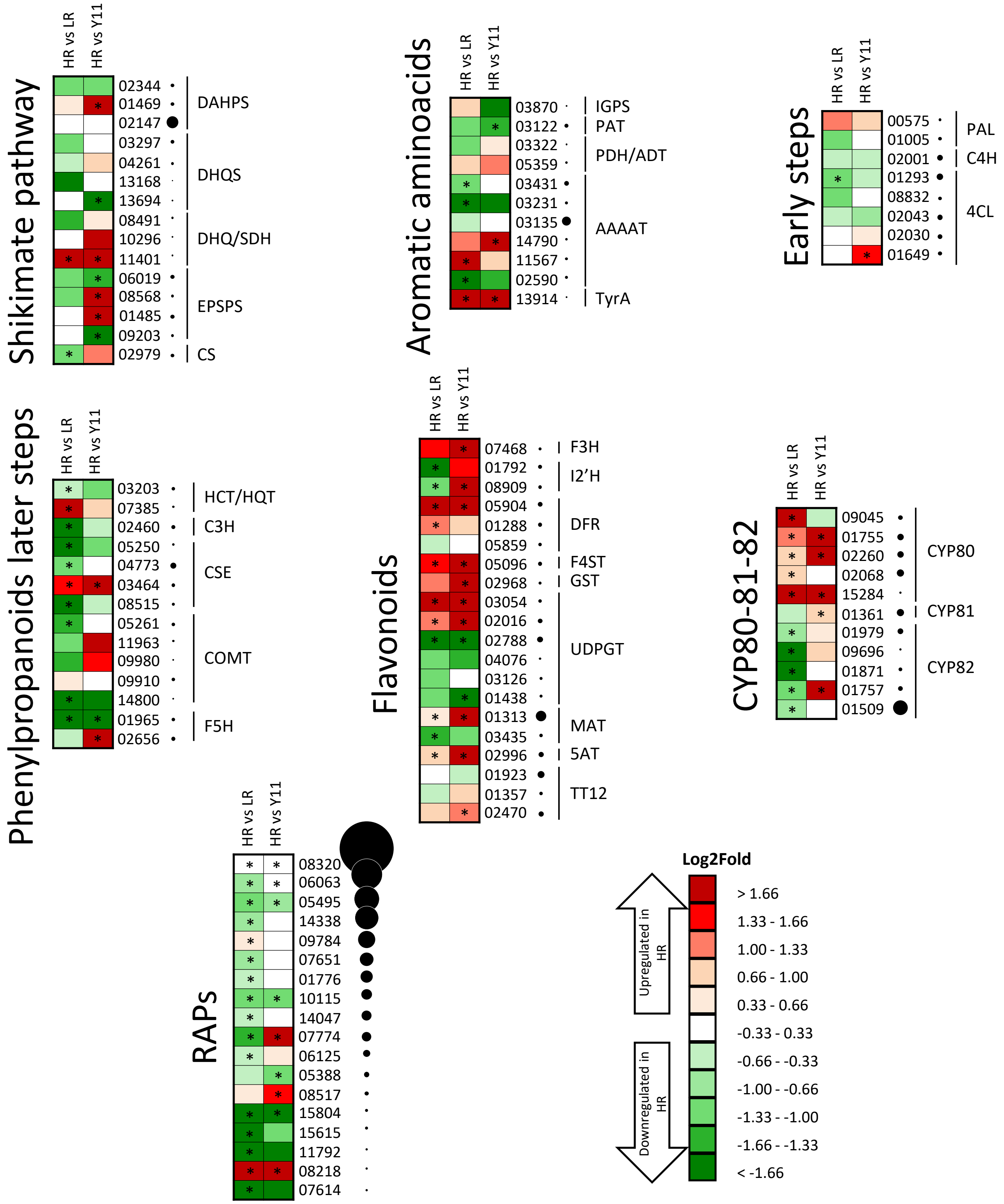

Figure S10. Expression of contigs involved phenylpropanoid and flavonoid biosynthesis, and belonging to CYP80-81-82 and RAP (Root allergen protein) categories in HR compared to LR and Y11 plants. Relative levels of expression are showed by a color gradient from low (green) to high (red). Asterisks indicate significant differences ($p < 0.05$). Black dots sizes are proportional to the number of reads. Abbreviations: DAHPS (3-deoxy-d-arabino-heptulosonate-7-phosphate synthase), DHQS (3-dehydroquianate synthase), DHQ/SDH (3-dehydroquianate dehydratase), EPSPS (5-enolpyruvylshikimate 3-phosphate synthase), CS (Chorismate synthase), IGPS (Indole-3-glycerol phosphate synthase), PAT (Prephenate aminotransferase), PDH/ADT (Prephenate dehydrogenase/arogenate dehydratase), AAAAT (Aromatic amino acid aminotransferase), TyrA (Arogenate dehydrogenase), PAL (Phenylalanine ammonia lyase), C4H (Cinnamate 4-hydroxylase), 4CL (4-coumarate:CoA ligase), HCT/HQT (Hydroxycinnamoyltransferase/Hydroxycinnamoyl-CoA: quinate hydroxycinnamoyltransferase), C3H (p-coumarate 3-hydroxylase), CSE (Caffeoyl shikimate esterase), COMT (Caffeate O-methyltransferase), F5H (Ferulate 5-hydroxylase), F3'H (Flavonoid 3'-hydroxylase), I2'H (Isoflavone 2'-hydroxylase), DFR (Dihydroflavonol-4-reductase), F4ST (Flavonol 4'-sulfotransferase), GST (Glutathione S-transferase), UDPGT (UDP-Glucosyltransferase), MAT (Anthocyanidin 3-O-glucoside-6"-O-malonyltransferase), 5AT (Anthocyanin 5-aromatic acyltransferase), TT12 (Transparent testa 12), RAP (Root allergen protein).
